# AID/APOBEC-like cytidine deaminases are ancient innate immune mediators in invertebrates

**DOI:** 10.1038/s41467-018-04273-x

**Published:** 2018-05-16

**Authors:** Mei-Chen Liu, Wen-Yun Liao, Katherine M. Buckley, Shu Yuan Yang, Jonathan P. Rast, Sebastian D. Fugmann

**Affiliations:** 1 Department of Biomedical Sciences, Chang Gung University, 259 Wenhua 1st Rd, Kwei-Shan District, Tao-Yuan, 333 Taiwan; 2 Biological Sciences, Sunnybrook Research Institute, 2075 Bayview Ave., Toronto, ON M4N 3M5 Canada; 3 Department of Medical Biophysics, University of Toronto, Toronto, MG5 1LC ON Canada; 4 Department of Immunology, University of Toronto, Toronto, M5S 1A8 ON Canada; 5 Division of Biochemistry, Molecular and Cellular Biology, Graduate Institute of Biomedical Sciences, Chang Gung University, Kwei-Shan District, Tao-Yuan, 333 Taiwan; 6 Department of Pathology, Chang Gung Memorial Hospital, Tao-Yuan, 333 Taiwan; 7 Division of Microbiology, Graduate Institute of Biomedical Sciences, Chang Gung University, Kwei-Shan District, Tao-Yuan, 333 Taiwan; 8 Chang Gung Immunology Consortium, Chang Gung Memorial Hospital and Chang Gung University, Kwei-Shan District, Tao-Yuan, 333 Taiwan; 9 Department of General Surgery, Chang Gung Memorial Hospital, Tao-Yuan, 333 Taiwan; 10 Present Address: Department of Biological Sciences, Carnegie Mellon University, 4400 Fifth Avenue, Pittsburgh, PA 15213 USA; 11 Present Address: Pathology & Laboratory Medicine, Emory University School of Medicine, 1462 Clifton Road, Atlanta, GA 30322 USA

## Abstract

In the course of both innate and adaptive immunity, cytidine deaminases within the activation induced cytidine deaminase (AID)/apolipoprotein B editing complex (APOBEC) family modulate immune responses by mutating specific nucleic acid sequences of hosts and pathogens. The evolutionary emergence of these mediators, however, seems to coincide precisely with the emergence of adaptive immunity in vertebrates. Here, we show a family of genes in species within two divergent invertebrate phyla—the echinoderm *Strongylocentrotus purpuratus* and the brachiopod *Lingula anatina*—that encode proteins with similarities in amino acid sequence and enzymatic activities to the vertebrate AID/APOBECs. The expression of these invertebrate factors is enriched in tissues undergoing constant, direct interactions with microbes and can be induced upon pathogen challenge. Our findings suggest that AID/APOBEC proteins, and their function in immunity, emerged far earlier than previously thought. Thus, cytidine deamination is probably an ancient innate immune mechanism that predates the protostome/deuterostome divergence.

## Abstract (web summary)

The AID/APOBEC cytidine deaminase family are nucleic acid editors, important for antigen receptor expression and thought to have evolved along with vertebrate adaptive immunity. Here the authors show this family may have evolved prior to adaptive immunity as members with cytidine deaminase activity are present and functional in invertebrate sea urchins and brachiopods.

## Introduction

Vertebrate immune systems have a range of defense mechanisms to target invading microorganisms, including directed mutagenesis of host and pathogen genomes and transcriptomes^1^. Activation induced cytidine deaminase (AID)/apolipoprotein B editing complex (APOBEC) family members participate as the corresponding mutator enzymes in both adaptive and innate immunity. The resulting mutations, which occur in both DNA and RNA molecules, have been implicated in restricting retroviruses and retrotransposons^2^, increasing transcriptional diversity in macrophages^3^, and diversifying antigen receptor repertoires^4^. The arguably most studied examples thereof are the AID-mediated changes to immunoglobulin genes during somatic hypermutation and class switch recombination^5–7^, and APOBEC3G restriction of HIV transcripts in humans^8–10^. To date, the most evolutionary ancient enzymes in this group that have been characterized are two cytidine deaminases (CDAs) within the agnathan lineage, which are involved in somatic rearrangement of the variable lymphocyte receptor genes^11^. The presence of these enzymes and related immune functions in both major vertebrate lineages suggests that an ortholog of the AID/APOBEC family was present in the last common vertebrate ancestor^12^. In previous attempts to discover if these enzymes predated the vertebrate lineage, similarity-based searches of invertebrate genome and transcriptome sequences have not identified convincing candidates. Consequently, an alternate strategy was employed to investigate whether related proteins are present in extant invertebrates. Here, an unbiased transcriptome analysis from sea urchin coelomocytes and a subsequent targeted search in a brachiopod results in the discovery of such AID/APOBEC homologs. Bacterial assays for mutator activity demonstrate conserved enzymatic functions and gene expression studies support a role in invertebrate immunity. Thus the role of AID/APOBEC in host defenses is much older than previously anticipated.

## Results

### Identification of AID/APOBEC-like transcripts in sea urchin

**Fig. 1:**
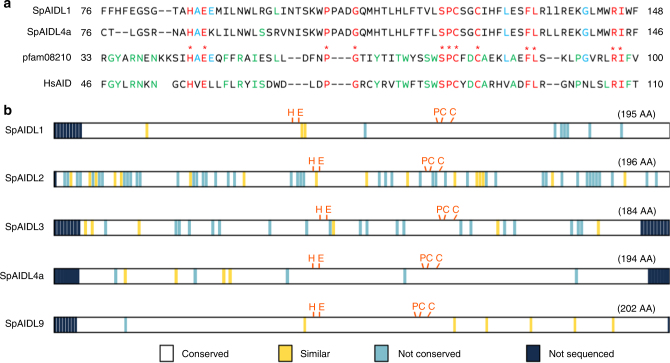
Sequence conservation of AID/APOBEC-like deaminases in *S. purpuratus*. **a** Amino acid sequence alignments of human AID (HsAID) and the *S. purpuratus* SpAIDL1 and SpAIDL4a with the pfam08210 APOBEC-like N-terminal domain present in all vertebrate AID/APOBEC polynucleotide deaminases. Absolutely conserved residues including the catalytic HxE-PCxxC motif are shown in red, while conservation limited to HsAID or SpAIDL1/4a and pfam08210 are indicated in green or blue, respectively. **b** Schematic representation of the open reading frames of SpAIDL1, 2, 3, 4a, and 9 encoded by alleles from three unrelated individuals. Regions where the PCR primers precluded obtaining sequences are marked in dark blue, and the location and nature of polymorphisms are indicated

RNAseq data generated from *Strongylocentrotus purpuratus* immune cells (coelomocytes) were used to assemble de novo transcriptomes. Comparison of these transcriptome data with the currently annotated *S. purpuratus* genome (*S. purpuratus* genome v3.1, www.echinobase.org^13^) reveals that many of the coelomocyte transcripts do not correlate with known gene models. Notably, two of these novel transcripts are predicted to encode proteins that contain an APOBEC-N domain (pfam08210), and are hereafter referred to as *S. purpuratus AID-like 1* and *4a* (*SpAIDL1* and *SpAIDL4a*; Genbank MH106904 and MH106905; Fig. 1). This domain, which is present in all vertebrate AID/APOBEC CDAs, is characterized by the conserved H[AVS]E-x(24-36)PCxxC motif that is essential for catalysis^14^. This catalytic motif is conserved within the SpAIDL1 and SpAIDL4a proteins, which are similar in size to human AID (HsAID, 198 AA; SpAIDL1, 193 AA; SpAIDL4a, 194 AA).

Phylogenetic analysis of the AID/APOBEC family members revealed that the SpAID proteins cluster with PmCDA2, an AID homolog from the sea lamprey *Petromyzon marinus* mediating the somatic assembly of variable lymphocyte receptor genes, at the base of the clade containing the vertebrate AID orthologs. Notably, throughout many iterations of alignments and tree-building, the SpAID proteins never formed a supported clade with the distantly related CDAs or deoxycytidine-5-monophosphate deaminases (dCMP DAs), although they share a highly similar catalytic motif (Supplementary Fig. 1). Putative nucleic acid deaminase proteins from *Nematostella vectensis* and *Caenorhabditis elegans* have been previously identified that exhibit extremely low similarity to the vertebrate AID orthologs^15^. Similar divergent factors have been predicted from automated scans of the *S. purpuratus* and *Crassostrea gigas* genomes, although some of these differ in size, represent incomplete fragments, or lacked one or more of the highly conserved residues^16,17^. None of these corresponded to the *SpAIDL* genes presented here and it remains unknown if any of these sequences are part of functional transcripts. We thus propose that the *SpAIDL* genes are the first reported invertebrate members of the AID/APOBEC family.

Among vertebrate species, the AID/APOBEC deaminase family exhibits different grades of expansion. To identify additional orthologs in *S. purpuratus*, the SpAIDL1 and SpAIDL4a sequences were used as queries in a tBLASTN search against the genome. Nine genomic loci (*SpAIDL1-9*) were identified that encode related proteins, including the source of our initial *SpAIDL1* transcript (98% identity to the ORF) (Supplementary Fig. 2). *SpAIDL4a* shows the highest similarity (91% nucleotide identity) to two loci, *SpAIDL4* and *SpAIDL5*. These are two of five predicted genes (*SpAIDL4-8*) that are inactive in the reference genome due to frame-shift mutations. Thus, the *SpAIDL4a* transcript may represent a functional allele of either *SpAIDL4* or *SpAIDL5*, but could also originate from a locus that is incorrectly assembled or absent from the current genome assembly. Interestingly, *SpAIDL1* and *SpAIDL2* are present as Gnomon predicted genes in Genbank (non-permanent accession numbers XM_011676409 and XM_011676403). We subsequently focused on the functional genes/transcripts: *SpAIDL1*, *2*, *3*, *4a*, and *9*. To determine the complete ORF of *SpAIDL9*, the full-length cDNA sequence of this transcript was obtained by 5′ and 3′ RACE (Genbank KY241384 and KY241385). Finally, to eliminate the possibility that the functional *SpAIDL* genes (*SpAIDL1*, *2*, *3*, *4a*, and *9*) are assembly artifacts, the coding regions of these sequences were isolated from the genome of three unrelated *S. purpuratus* individuals using PCR (Fig. 1b). Analysis of these sequences indicate the existence of numerous alleles in the *S. purpuratus* population, and interestingly numerous allelic variations of the anti-viral APOBEC3H deaminase have been reported in the human population^18,19^.

### Expression patterns of SpAIDLs

**Fig. 2:**
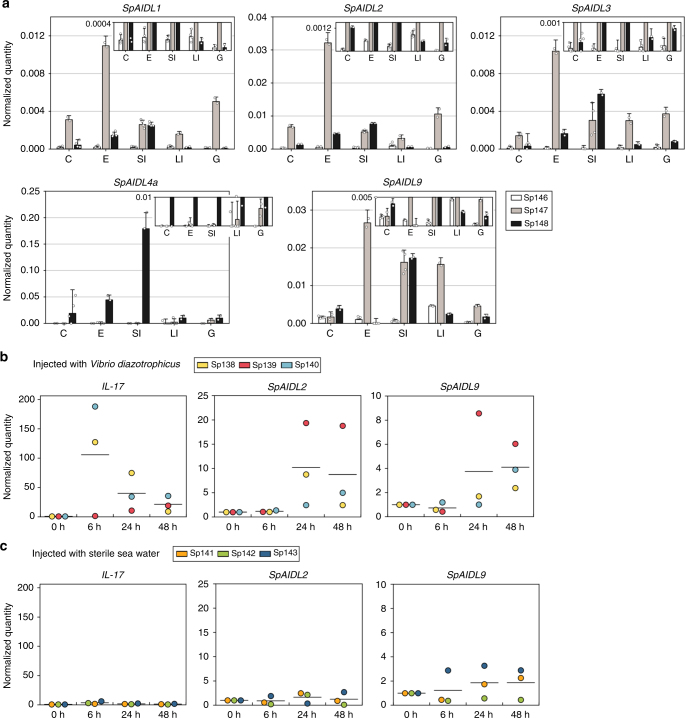
Expression of *SpAIDLs* in *S. purpuratus*. **a** The expression levels of *SpAIDL1, 2, 3, 4a, 9*, and *18S* RNAs in coelomocytes (C), the esophagus (E), the small intestine (SI), the large intestine (LI), and the gonad (G) obtained from three individual *S. purpuratus* (Sp146♀, Sp147♂, and Sp148♀) were measured by RT-qPCR with two technical replicates per sample. The expression levels are normalized to the amounts of *18S*, and the error bars indicate the standard deviation. The insets are a magnified view. **b**, **c** Six animals were injected with either live *V. diazotrophicus* (Sp138, 139, and 140) or sterile sea water (Sp141, 142, and 143). The time course of *SpAIDL2*, *SpAIDL9*, and *SpIL17-9* expression levels were measured by RT-qPCR with each data point being the mean of two technical replicates. The expression levels were first normalized to *18S* and then to the expression at *t* = 0 for each transcript for each sea urchin. The horizontal bars indicate the mean

Unlike most of the vertebrate AID/APOBEC deaminases that are broadly expressed^20^, AID expression is restricted to specific tissues^21^. To localize *SpAIDL* gene expression, tissues were collected from three sea urchins (animals Sp146, Sp147 and Sp148), and transcript levels were quantified by RT-qPCR (Fig. 2a). Results indicate that the *SpAIDL* genes are highly regulated: transcript levels varied widely among individuals and some transcripts were exclusive to or absent from one animal (e.g., *SpAIDL4a* in Sp148 and *SpAIDL3* in Sp146). Although *SpAIDL* transcripts were detected in all tissues, the highest expression levels were evident within the digestive tract (esophagus, small intestine, and large intestine; Fig. 2a). Notably, *SpAIDL1* and *SpAIDL4a*, which were initially recovered from coelomocytes, were expressed at low levels in these echinoderm immune cells in these seemingly healthy, uninfected individuals. The tissues of the digestive system are populated with large numbers of resident coelomocytes and are in constant, close proximity to the lumenal microbiota of the digestive tract. The SpAIDL proteins may thus function at these sites to maintain commensals and to combat pathogens, similar to the specialized, gut-associated immune cells in vertebrates. This highly regulated expression and the preferential presence of *SpAIDL1, 2, 3, 4a*, and *9* transcripts in the digestive system are consistent with a potential function of the encoded proteins in immunity.

To determine whether the transcription of the *SpAIDL* genes is induced in response to immune challenge, we utilized a bacterial infection model using *Vibrio diazotrophicus* (Fig. 2b, c). This gram-negative bacterium was originally isolated from the gut of an adult sea urchin^22^, and activates immune cells in *S. purpuratus* larvae^23^ and adults^24^. Three individual sea urchins were injected with either a single dose of live *V. diazotrophicus* or a sterile sea water control. As expected, the bacteria induced an immune response: within 6 h, *SpIL17-9*, an early marker of immune activation in *S. purpuratus* coelomocytes^25^ was upregulated in the *Vibrio*-infected individuals. Notably, bacterial challenge also resulted in the increased levels of *SpAIDL2* transcripts after 24 h of infection, whereas no change was observed in the control cohort (Fig. 2b, c). This mirrors the induction of *aicda* expression during B cell responses in mice^5^. Intriguingly, while changes in the expression of *SpAIDL9* and all other *SpAIDL* genes were also observed, neither showed a pattern that strictly correlated with the *Vibrio* infection in all sea urchins (Fig. 2b and Supplementary Fig. 3). The basis for this variability remains elusive and may be due to differences in genotype, sex, and immune status of individual animals. Given the role of APOBEC3 paralogs in anti-viral responses^2^, it is conceivable that infection with other pathogens in particular viruses may upregulate other SpAIDL family members. Overall our expression data is consistent with a role of the *SpAIDL* genes during host–microbe interactions in *S. purpuratus*.

### Enzymatic activities of SpAIDLs

**Fig. 3:**
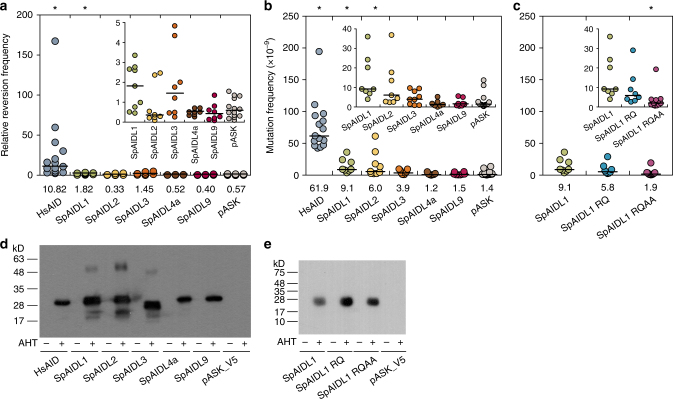
Enzymatic activities of SpAIDLs. **a**–**c** Deaminase activities of each indicated protein were measured in bacterial reversion assays using either a Kanamycin resistance reporter plasmid (**a**) or the endogenous rpoB gene as the reporter (**b**, **c**). Note that the SpAIDL1 dataset from **b** is shown again in **c** for clarity. The horizontal bars represent the median (numerical value below each column), and *p*-values (for each deaminase compared to empty pASK for **a**, **b**, and compared to wildtype SpAIDL1 for **c**) were calculated using a Willcoxon rank sum for unpaired data. Datasets with *p* < 0.05 are marked with an asterisk (**a**: HsAID *p* = 0.000001 and SpAIDL1 *p* = 0.02087; **b**: HsAID *p* = 6.6 × 10^−9^, SpAIDL1 *p* = 0.0005, and SpAIDL2 *p* = 0.0014; **c**: SpAIDL1 RQAA *p* = 0.00048). The inset represents a magnified view omitting all data points of HsAID (**a**, **b**) and one datapoint of SPAIDL2 (**b**) for clarity. SpAIDL1 RQ and SpAIDL1 RQAA are mutants of SpAIDL1 in which either two (H86R and E88Q) or four (H86R, E88Q, C123A, and C126A) of the conserved active site residues were altered. **d**, **e** Western blot analysis of the expression of the indicated V5-tagged deaminases in bacteria induced with AHT for 3 h (+) or not (−). Note that the enzymatic activities were tested with untagged versions of the proteins. The empty pASK_V5 plasmids served as a control. The predicted sizes for V5-tagged HsAID, SpAIDL1 (and the mutants thereof), 2, 3, 4a, and 9 are 25.4, 24.0, 23.5, 22.3, 23.6, and 24.6 kD, respectively

The vertebrate AID/APOBEC proteins mediate anti-pathogen responses by deaminating cytosine to uracil, thus altering the nucleotide sequence of DNA or RNA molecules^1^. To determine if the SpAIDL proteins exhibit similar enzymatic activity, we employed an *E. coli*-based colony formation assay that is routinely used to assess the activities of vertebrate deaminases^26^. Individual SpAIDL proteins were expressed in *E. coli* containing a mutated kanamycin resistance reporter gene (Kan^R^ L94P). AID/APOBEC-like deaminase activity reverses this mutation, conferring kanamycin resistance in individual bacterial cells. In this assay, HsAID and SpAIDL1 showed significantly higher reversion frequencies compared to the empty expression vector (pASK) controls (HsAID *p* = 0.000001 and SpAIDL1 *p* = 0.02087; *p* < 0.05; Fig. 3a). SpAIDL3 also exhibited higher reversion rates than controls, although this did not reach statistical significance (*p* = 0.051). Importantly, in parallel experiments, V5-tagged versions of all proteins were expressed at comparable levels in *E. coli* (Fig. 3d and Supplementary Fig. 4). The Kan^R^ reporter-based deaminase assay relies on the reversion of a single point mutation; it is thus possible that the target cytosine resides in a local sequence context that is unfavorable for the SpAIDL proteins, leading to very low or undetectable deaminase activity. Hence, the mutator activity of all SpAIDL proteins was also tested using the endogenous *E. coli rpoB* gene as a reporter (Fig. 3b). Several individual point mutations within this gene give rise to rifampicin resistance offering a wider range of substrates for the deaminases^27^. In this assay, HsAID, SpAIDL1, and SpAIDL2 exhibited significantly higher mutation frequencies compared to the empty expression vector (pASK) control (*p* = 6.6 × 10^−9^, *p* = 0.0005, and *p* = 0.0014, respectively). As in the Kan^R^ assay, SpAIDL3 activity was higher than the control, but did not reach statistical significance (*p* = 0.17).

The apparent lack of activity for the remaining SpAIDL paralogs in both assays may reflect the non-physiological context in which the experiments are performed. Particularly relevant factors are the temperature, which is much higher under experimental (37 °C) than under native (15 °C) conditions, and the salt concentration which is much lower than in the native marine environment. Notably, deaminase function is not essential for all vertebrate APOBEC3 immune functions^28^. The absence of enzymatic activity may therefore reflect an independent immune-related function for these sea urchin proteins. Finally, these paralogs may represent homologs of vertebrate APOBEC2/APOBEC4, the two AID/APOBEC family members for which clear enzymatic activity and molecular function have not yet been assigned, and thus an assay has not been designed to assess their functionality^14^.

**Fig. 4:**
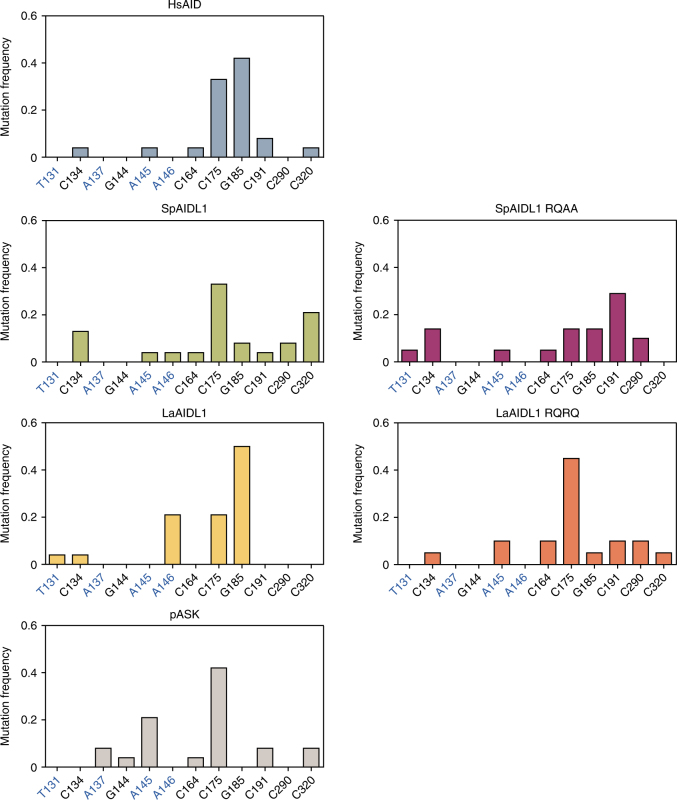
Mutation patterns generated by SpAIDL1 and LaAIDL1. Genomic DNA fragments of the *rpoB* gene from 24 individual Rif^R^ colonies from the *E. coli*-based deaminase assay (Figs. 3, 5) were amplified by PCR and sequenced. For each deaminase a minimum of 20 interpretable sequences were obtained (HsAID: 24, SpAIDL1: 24, SpAIDL1 RQAA: 21, LaAIDL1: 24, LaAIDL1 RQRQ: 20, pASK: 24). The frequencies of mutations at individual nucleotides numbered relative to the 5′-end of the PCR product (with A/Ts shown in blue) are shown as bar graphs. Colonies from bacteria containing only the empty expression vector (pASK) served as the negative control for spontaneously arising mutations conferring resistance to rifampicin

To further characterize the deaminase activity of SpAIDL1 we determined the nucleotide changes that gave rise to rifampicin resistance in *E. coli* expressing SpAIDL1, HsAID, or containing the empty expression vector (pASK) (Fig. 4). SpAIDL1-dependent mutations occurred largely at G or C residues (96%). The profile of these mutations, which clearly differed from both those introduced by HsAID and the spontaneously arising background mutations, is consistent with specific cytosine deaminase activity. The catalytic site of the vertebrate AID/APOBEC proteins has been well characterized and is required for enzymatic function. To assess the role of the four residues that comprise this active site (which are conserved among all vertebrate AID/APOBECs and SpAIDLs), we generated two mutants: SpAIDL1 RQ and SpAIDL1 RQAA, which contained mutations in either two (H86R and E88Q) or all four residues (H86R, E88Q, C123A, and C126A). While SpAIDL1 RQ showed only a modest reduction in activity (Fig. 3c), SpAIDL1 RQAA activity was almost undetectable and significantly lower (*p* = 0.00048) than that of the wildtype protein despite comparable expression of respective V5-tagged proteins in parallel experiments (Fig. 3c, e, and Supplementary Fig. 4). Strikingly, the profile of *rpoB* mutations observed in *E. coli* expressing the SpAIDL1 RQAA mutant clearly differs from that of the wild-type protein, which is consistent with the dramatic reduction in the deaminase activity (Fig. 4). Together, these data indicate that, similar to many vertebrate AID/APOBEC factors, at least two of the SpAIDL proteins are able to deaminate cytosines within ssDNA in these assays. This AID/APOBEC enzymatic function thus predates the emergence of the vertebrates.

### AID/APOBEC-like deaminases in a brachiopod

**Fig. 5:**
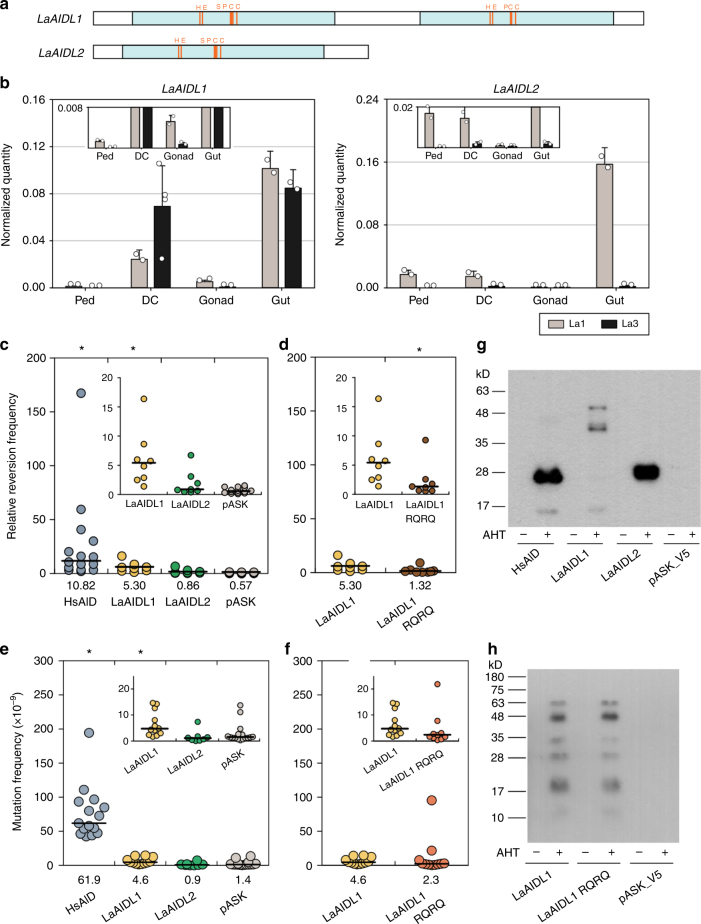
Characterization of LaAIDLs in *L. anatina*. **a** Schematic view of LaAIDL1 and 2 with deaminase domains shown in light blue and the catalytic residues in red. **b** Normalized expression levels of *LaAIDL1, 2*, and *LaRPL39* from the pedicle (Ped), the digestive cecum (DC), the gonad, and the gut from two individuals (La1 and La3) were measured by RT-qPCR with two technical replicates per sample. The error bars indicate the standard deviation. The insets represent magnified views. **c**–**f** Deaminase activities of LaAIDL1, LaAIDL2, and mutants of the former were measured in bacterial reversion assays using either a Kanamycin resistance reporter plasmid (**c**, **d**) or the endogenous rpoB gene as the reporter (**e**, **f**). LaAIDL1 RQ RQAA is putative catalytic mutant in which two residues in both active sites (H81R, E83Q, H298R, and E300Q) were altered. The data for HsAID and empty pASK plasmids is the same as shown in Fig. 3. The horizontal bars represent the median (value below each column), and *p*-values were calculated for each deaminase compared to empty pASK (**c**, **e**), and compared to wildtype LaAIDL1 for (**d**, **f**) using a Willcoxon rank sum test for unpaired data. Datasets with *p* < 0.05 (**c**: HsAID *p* = 0.000001 and LaAIDL1 *p* = 0.000053; **d**: LaAIDL1 RQRQ *p* = 0.021; **e**: HsAID *p* = 6.6 × 10^−9^ and LaAIDL1 *p* = 0.0022) are marked with asterisks. The insets represent a magnified views of the same data omitting all data points of HsAID (**c**, **e**) and one data point of LaAIDL1 RQRQ (**f**) for clarity. **g**, **h** Bacterial expression of V5-tagged LaAIDL1 and 2 (and mutants thereof), and HsAID in *E. coli* was assessed using a monoclonal anti-V5 antibody (for details see legend to Fig. 3b). Note that the enzymatic activities were tested with untagged versions of the proteins. The predicted sizes for V5-tagged HsAID, LaAID1, and 2 are 25.4, 52.4, and 23.5 kD, respectively, and smaller bands likely represent degradation products

To determine if these AID/APOBEC-like deaminases arose prior to the last common ancestor of deuterostomes, the SpAIDL proteins were used to query protostome genome sequences with tBLASTN. Two hits emerged from the genome and transcriptome of the brachiopod *Lingula anatina*, hereafter referred to as *L. anatina* AID-like 1 and 2 (*LaAIDL1* and *LaAIDL2*). These genes encode proteins with two striking features: (1) LaAIDL1 contains two tandem deaminase domains (Fig. 5a), which resembles the structure of human APOBEC3B/D/F/G; and (2) in BLASTP searches using LaAIDL1 and LaAIDL2 as queries, avian and primate APOBEC1 and APOBEC3G proteins were amongst the top matches (Tables 1 and 2). As in sea urchins, *LaAIDL1* and *LaAIDL2* transcripts were enriched in tissues of the digestive system in adult *L. anatina* (Fig. 5b). The *LaAIDL2* transcripts were detectable in only one of the individuals, suggesting that this gene may be tightly regulated (Fig. 5b). This differential regulation and expression in tissues with persistent host–microbe interactions point to a function in immune response.

**Table 1:** Genbank entries with the highest similarity to LaAIDL2 (BLASTP)

Genbank acc#	Species	Protein name
XP_013401343.1	*Lingula anatina*	PREDICTED: uncharacterized protein LOC106167175^a^
XP_013387831.1	*Lingula anatina*	PREDICTED: uncharacterized protein LOC106156932
XP_019725238.1	*Hippocampus comes*	PREDICTED: uncharacterized protein LOC109515716 isoform X1
XP_019725239.1		PREDICTED: uncharacterized protein LOC109515716 isoform X2
XP_010387566.1	*Rhinopithecus roxellana*	PREDICTED: DNA dC->dU-editing enzyme APOBEC-3H isoform X2
XP_010387568.1		PREDICTED: DNA dC->dU-editing enzyme APOBEC-3H isoform X3
XP_010387564.1		PREDICTED: DNA dC->dU-editing enzyme APOBEC-3H isoform X1
XP_011451320.2	*Crassostrea gigas*	PREDICTED: uncharacterized protein LOC105345037
XP_017715376.1	*Rhinopithecus bieti*	PREDICTED: DNA dC->dU-editing enzyme APOBEC-3H isoform X3
XP_017715377.1		PREDICTED: DNA dC->dU-editing enzyme APOBEC-3H isoform X4
XP_015272576.1	*Gekko japonicus*	PREDICTED: DNA dC->dU-editing enzyme APOBEC-3G-like
XP_012966578.1	*Mesocricetus auratus*	DNA dC->dU-editing enzyme APOBEC3-like

^a^Now referred to as LaAIDL2

**Table 2:** Genbank entries with the highest similarity to LaAIDL2 (BLASTP)

Genbank acc#	Species	Protein name
XP_013401332.1	*Lingula anatina*	PREDICTED: uncharacterized protein LOC106167165^a^
XP_014795583.1	*Calidris pugnax*	PREDICTED: C->U-editing enzyme APOBEC-1-like isoform X2
XP_009483208.1	*Pelecanus crispus*	PREDICTED: C->U-editing enzyme APOBEC-1-like
XP_019924665.1	*Crassostrea gigas*	PREDICTED: uncharacterized protein LOC109619300
EMC82544.1	*Columba livia*	C->U-editing enzyme APOBEC-1, partial
OPJ69246.1	*Patagioenas fasciata monilis*	C->U-editing enzyme APOBEC-1
XP_010157863.1	*Eurypyga helias*	PREDICTED: C->U-editing enzyme APOBEC-1-like

^a^Now referred to as LaAIDL1

To assess the potential enzymatic activity of LaAIDL1 and LaAIDL2, the *E. coli*-based Kan^R^ and *rpoB* deaminase assays described above were employed. Strikingly, the median reversion frequency of LaAIDL1 was 49% of that observed for HsAID (Fig. 5c). This is particularly notable given the reduced protein expression level (Fig. 5g and Supplementary Fig. 4), suggesting that the specific activity of LaAIDL1 may be greater than that of HsAID. Deaminase activity of LaAIDL1 was also observed in the endogenous *rpoB* gene (Fig. 4e, *p* = 0.0022) but only at 10% of that for HsAID. Mutating two conserved residues in each of the putative active sites (LaAIDL1 contains two APOBEC domains; LaAIDL1 RQRQ) reduced overall enzymatic activity (Fig. 5d, f) albeit being only statistically significant in the KanR reversion assay (*p* = 0.021), and drastically altered the *rpoB* mutation pattern in rifampicin-resistant *E. coli* expressing LaAIDL1 RQRQ such that it closely resembled that of control bacteria (Fig. 4). Together, these observations suggest that functional AID/APOBEC-like deaminases were present in the last common bilaterian ancestor. Furthermore, the conserved expression patterns and enzymatic activities of these newly described invertebrate AID/APOBEC proteins are consistent with an ancient function in immune response.

## Discussion

The most parsimonious explanation for the broad evolutionary distribution of AID/APOBEC-like CDAs within the bilaterians is that these proteins evolved from a common ancestor. The data presented here suggest that at least some aspects of immune function exists among these orthologs. Specifically, as in vertebrates, *SpAIDL* and *LaAIDL* transcripts are enriched in immunologically active tissues. Furthermore, we observe a clear upregulation of *SpAIDL2* transcripts in response to immune activation, which mirrors the behavior of *aidca* in activated B cells. Finally, at least some members of the SpAIDL/LaAIDL deaminases exhibit catalytic activity against polynucleotide substrates, that differs from background mutation profiles and is dependent on specific residues within an active site that is conserved across all vertebrate and invertebrate members of the AID/APOBEC deaminase family. Although specific activities and transcriptional regulation vary among these invertebrate AID/APOBEC-like enzymes, this is consistent with specialized functions of the vertebrate orthologs and suggests that the broader immune function is an ancient feature of this protein family.

What might be the molecular targets of the SpAIDL and LaAIDL enzymes? The vertebrate AID/APOBEC family can be subdivided into polynucleotide deaminases that exhibit mutator activities in the *E. coli*-based assay (AID, APOBEC1, APOBEC3, and *Petromyzon marinus* CDA1 and CDA2) and those that are either inactive in this experimental context or act on yet to be discovered substrates (APOBEC2 and APOBEC4)^14^. Given the similarity to the vertebrate enzymes, it is tempting to speculate that the “active” invertebrate deaminases represent functional homologs of the former acting on diverse targets ranging from foreign DNA, including viral genomes and replication intermediates, to host cell genomic DNA and transcripts, while the “inactive” SpAIDLs and LaAIDL2 might serve similar roles to the latter. In the bacterial mutator assay, anti-foreign DNA APOBECs typically show higher activities than AID^27^, whose cognate targets are the endogenous immunoglobulin genes. This raises the possibility that SpAIDL1 and LaAIDL1 (and potentially SpAIDL2 and SpAIDL3) might actually edit genes or transcripts important for invertebrate immune responses. But it is equally conceivable that the current assay is simply not optimized to reveal the full enzymatic potential of polynucleotide deaminases from marine invertebrates.

Even in the highly unlikely scenario that future, in-depth studies of the novel invertebrate AID/APOBEC-like proteins do not fully corroborate their currently predicted immune functions, the mere existence of invertebrate AID/APOBEC deaminase family members has important implications for understanding the evolution of innate and adaptive immunity. Altering exogenous genetic material is an ancestral principle of anti-viral immunity that is present even in bacteria that rely on restriction enzymes and CRISPR/Cas9 systems to detect and digest phage DNA^29,30^. Hence, we favor a model in which an ancestral AID/APOBEC enzyme in invertebrates acted on non-self DNA; this central function has been retained throughout evolutionary time. The evolutionary pressures from species-specific pathogens shaped a divergent repertoire of these powerful mutator enzymes, which is exemplified in the distinct anti-viral specificities of mammalian APOBEC3 proteins^31,32^. Co-opting and redirecting the mutagenic activity of one AID/APOBEC family member toward endogenous genes in an ancestor of vertebrates, and restriction of its expression to the primordial lymphocyte may have led to the emergence of one of the unique and central features of vertebrate adaptive immunity: highly diversified antigen receptor repertoires that mediate pathogen recognition. The critical enzymes underlying this somatic diversification are AID, which directs somatic hypermutation and class switch recombination in jawed vertebrates and its jawless vertebrate homologs CDA1 and CDA2, which are involved in rearrangement of the variable lymphocyte receptor genes. Strikingly, this evolutionary trajectory is shared with V(D)J recombination the other somatic gene diversification process in jawed vertebrates^33,34^. Together these findings imply that adaptive immunity arose by fundamental principles of evolution: shuffling, re-purposing, and integration of pre-existing factors and processes.

## Methods

### Animals

*S. purpuratus* were maintained in artificial sea water at 14 °C. *L. anatina* were collected in June 2016 in the Xiangshan wetlands, Xiangshan District, Hsinchu City, Taiwan (GPS coordinates 24.770779, 120.910742 E), transported to the laboratory on ice, and then dissected immediately.

### Constructs and plasmids

To determine the enzymatic activity of SpAIDLs and LaAIDLs, respective bacterial expression vectors pASK-SpAIDLX/LaAIDLX/HsAID were generated for SpAIDL1, 2, 3, 4a, 9, LaAIDL1, 2 and HsAID. The open reading frames of each *SpAIDL* were amplified from genomic DNA by PCR using Phusion polymerase and primers containing SpeI and XhoI restriction sites (Supplementary Table 1). The PCR products were digested with SpeI and XhoI (New England Biolabs) and inserted into the NheI/XhoI sites of pASK-TadA (a gift from Dr. Nina Papavasiliou, Rockefeller University, New York) replacing the TadA open reading frame. For SpAIDL9 this cloning strategy changed the third amino acid from phenylalanine to serine, and this F3S mutation was reverted back to F3 using the Quikchange mutagenesis kit (Stratagene) with primers SPA9S3FT/SPA9S3FB (Supplementary Table 1). *HsAID* was amplified from a plasmid containing human full length AID (pCDF-AID, a gift from Dr. Nina Papavasiliou, Rockefeller University, New York). The cloning strategy changed the second amino acid from aspartate to alanine, and D2A was reverted back to D2 using the Quikchange mutagenesis kit (Stratagene) with primers hAIDA2DT/hAIDA2DB (Supplementary Table 1). The empty pASK was generated by NheI/XhoI digestion of pASK-TadA, filling in the overhangs using Klenow polymerase, and ligation with T4 DNA ligase (Thermo Scientific). The open reading frames of each *LaAIDL* were amplified from *L. anatina* cDNA and cloned into the pJET1.2/blunt cloning vector (Thermo Scientific). The *LaAIDLX* open reading frames were excised with SpeI and XhoI (New England Biolabs), and cloned into the XbaI/XhoI sites of pASK_V5-SpAID4a (described below), replacing the SpAID4a insert. It should be noted that cloned fragments contain the original stop codons at their 3′ ends such that the expressed protein does not contain a V5 tag.

The expression constructs for the active site mutants of SpAIDL1 and LaAIDL1 (SpAIDL1 H86R E88Q, SpAIDL1 H86R E88Q C123A C126A, and LaAIDL1 H81R E83Q H298R E300Q) were generated by site-directed mutagenesis from the respective wild-type (or single mutant) plasmids using either the Quikchange mutagenesis kit (SPA1H85RE87QT/SPA1H85RE87QB for SpAIDL1 RQ), or by conventional PCR using 5′-phosphorylated primers (SPA1CCAA-P/SPA1CCAAB for the second set of alterations in SpAIDL1 RQAA, and LaA1H81RE83QB/LaA1H81RE83Q-P and LaA1H298RE300QB/LaA1H298RE300Q-P for SpLaAIDL1 RQRQ) followed by DpnI digestion, ligation to circularize the products, and transformation into competent *E. coli*.

To monitor the level of recombinant protein expression in *E. coli*, the bacterial expression constructs pASK_V5-SpAIDLX/LaAIDLX/HsAID were created. pASK_V5 was generated by annealing the oligonucleotides pASK_V5F/pASK_V5R (Supplementary Table 1) containing the V5 tag sequence, and ligating them into the NheI/SalI sites of pASK-TadA. The pASK-SpAIDLX/LaAIDLX plasmids were used as templates to amplify the respective cDNAs without stop codon, using Phusion polymerase and the primers listed in Supplementary Table 1, and cloned into the NheI/XhoI sites (for *SpAIDLX* and *HsAID* fragments) or XbaI/XhoI sites (for *LaAIDLX* fragments) of pASK_V5. *HsAID* was amplified from pASK-hAID. The sequences of both, *SpAIDL9* and *HsAID*, were again changed by this cloning strategy, and were changed back to the correct sequences as described above.

### Genomic DNA preparation

Genomic DNAs of both, *S. purpuratus* and *L. anatina*, were purified using the gDNA Spin Kit Tissue (Bioman) following the manufacturer’s instructions. Briefly, approximately 1 × 10^6^ *S. purpuratus* coelomocytes and 100 mg *L. anatina* tissue were added to separate microcentrifuge tubes, homogenized in 200 µl buffer GT (Bioman) containing 200 mg of Proteinase K, and incubated for 30 min at 60 °C to completely lyse the cells. After addition of 200 µl BG buffer (Bioman), the samples were vortexed and incubated for 20 min at 70 °C. Subsequently 200 µl ethanol were added and the entire sample was loaded onto a GD spin column (Bioman). Washes with 400 µl of WI buffer (Bioman) and 600 µl wash buffer were performed by centrifugation at 10,000×*g* for 30 s. This was followed by elution of the genomic DNAs with 100 µl of elution buffer (10 mM Tris pH = 8.0) that was pre-warmed to 60 °C.

### RNA preparation and cDNA synthesis

*S. purpuratus* coelomic fluid was drawn with a syringe through the periostomal membrane and mixed immediately with an equal volume of CMSFW-EI (0.53 M NaCl, 10 mM KCl, 2.4 mM NaHCO_3_, 11 mM Na_2_SO_4_, 30 mM EDTA, 50 mM imidazole). The coelomocytes were spun down and resuspended in 800 μl EasyPure Total RNA Reagent (Bioman). The esophagus, small intestine, large intestine, and gonads from *S. purpuratus* and the pedicle, digestive cecum, gonad and gut from *L. anatina* were dissected from individual animals. Small pieces of the *S. purpuratus* and *L. anatina* solid tissues were added to 200 μl EasyPure Total RNA Reagent (Bioman), and the samples were homogenized in a Bullet Blender (Next Advance) using 0.5 mm ZrO beads. Insoluble debris was spun down and the supernatants were transferred to fresh tubes containing 600 μl EasyPure Total RNA Reagent (Bioman) and 200 μg glycogen as a carrier. Total RNA was then purified according to the manufacturer’s protocol. To remove the residual gDNA and other contaminants, *S. purpuratus* RNA were then further processed using either the Turbo DNA-free kit (Ambion) or the RNeasy mini kit (Qiagen). The Turbo DNA-free kit (Ambion) was used according to the manufacturer’s protocol, except that extra MgCl_2_ (2.5 mM) and CaCl_2_ (1 mM) were added for the DNase digestion. The RNeasy mini kit (Qiagen) was used according to the manufacturer’s protocol.

Approximately 250–500 ng of *S. purpuratus* and *L. anatina* RNA from each tissue were converted to cDNA using the ThermoScript RT-PCR System (Invitrogen) or the ToolsQuant II Fast RT Kit (Biotools), respectively. In both cases random hexamers were used and the manufacturer’s instructions were followed.

### Rapid amplification of cDNA ends

The 5′ and 3′ ends of the SpAIDL9 transcripts were amplified using the SMARTer RACE kit (Clontech) and the gene specific primers SPA9CR1, SPA9CR2, SPA9CF1, SPA9CF2 (see Supplementary Table 2) according to the manufacturer’s instructions. PCR products containing the 5′ and 3′ ends of the transcripts were cloned into the pJET1.2/blunt cloning vector (Thermo Scientific), and individual clones were sequenced entirely.

### Sequence analysis of the *SpAIDL* genes

Genomic DNA from three *S. purpuratus* individuals (Sp105, Sp111, Sp112) were used as templates to amplify the open reading frames of *SpAIDL1, 2, 3, 4a, 9* using Phusion polymerase and the primers listed in Supplementary Table 2. The PCR products were cloned into the pJET1.2/blunt cloning vector (Thermo Scientific). Plasmids were purified using the Tools mini plasmid kit (Biotools) and submitted to a commercial service (Genomics Taiwan) for sequencing using either the pJET1.2 forward or the pJET1.2 reverse primers.

At least eight sequences were collected for each gene from each sea urchin, and the primer sequences were excluded for prior to the sequence alignments. The nucleotide sequences for each gene were first aligned within each *S. purpuratus* using CLUSTALW (www.genome.jp/tools/clustalw), and the dominant sequences corresponding to the two alleles were then selected for subsequently comparison between individual sea urchins.

### Gene expression analysis

Complementary DNA from three individual *S. purpuratus* (Sp146, Sp147, Sp148) and two individual *L. anatina* (La1 and La3) were used as templates to test the expression of either *SpAIDL1*, *2*, *3*, *4a*, and *9*, or *LaAIDL1* and *2* by quantitative PCR. Each qPCR reaction (10 μl) contained 5 μl Fast SYBR Green Master Mix (Applied Biosystems), primers (0.1 μM) (see Supplementary Table 3) and 1 μl of template, and for each sample two technical replicates were setup. The qPCR reactions were performed in a 7500 Fast Real-Time PCR System (Applied Biosystems), applying an initial denaturation step at 95 °C for 20 s followed by 40 cycles of 3 s at 95 °C and 30 s at 60 °C. Standard curves were used to determine gene expression levels and housekeeping genes (*Sp18S* for *S. purpuratus* and *LaRPL39* for *L. anatina*) were used for normalization.

### Bacterial infection model

*Vibrio diazotrophicus* were grown in Difco marine broth (Becton Dickinson) liquid cultures at 30 °C and 250 rpm overnight. Six healthy sea urchins were randomly selected and divided into two groups, an experimental group and a control group. Aliquots of coelomic fluid (0.5 ml) were drawn from each animal at *t* = 0. Sea urchins in the experimental group were injected with a suspension of live *Vibrio diazotrophicus* in 200 µl sterile artificial sea water with the amount of bacteria adjusted for each sea urchin to correspond to 10^6^ cells/ml of total coelomic fluid volume. The control group was injected with 200 µl sterile sea water. At *t* = 6, 24, and 48 h, 200 µl coelomic fluid was drawn from each animal in each group. RNA was extracted from the coelomocytes obtained at each time point, and from the *t* = 0 samples genomic DNA was prepared as well. The primer pairs for gene expression analysis (Supplementary Table 3) were first tested on the genomic DNA samples to ensure they can amplify the respective gene from each individual, despite the notable levels of polymorphism levels in their sequence within the *S. purpuratus* population (see Fig. 1b). Quantitative RT-PCR (as described above) was used to measure gene expression levels with at least two technical replicates per sample. Expression values were first normalized to the levels of 18S in each sample, and then to the values at *t* = 0 for each animal. Three biological replicates were used for the experimental and control group, and all data points are shown.

### CDA assays with kanamycin resistance reporter

An optimized protocol of a widely used assay to measure polynucleotide CDA activities^26^ was used. The pTAC-Kan deaminase reporter plasmid containing a L94P point mutation in the Kan^R^ gene and individual pASK-SpAIDLX/LaAIDLX expression vectors were transformed sequentially into the UNG-deficient BH156 *E. coli* strain. The human AID expression vector pASK-HsAID and the empty pASK vector served as positive and negative controls, respectively. For the deaminase assay, at least eight individual colonies for each putative deaminase were grown in LB broth (containing 50 μg/ml of ampicillin (Amp), 50 μg/ml of spectinomycin (Spc), and 17 μg/ml of chloramphenicol (Cam)) until the optical density at 600 nm reached 0.3. Each culture was then split in half. While IPTG (1 mM) was added to both of them, anhydrotetracycline (AHT) (0.2 μg/ml) was added only to one of them to induce the expression of SpAIDLX/LaAIDLX. After incubation for 3 h at 37 °C and 200 rpm, the cultures were spun down, washed with PBS, and spread onto LB plates containing either 50 μg/ml of Amp, 50 μg/ml of Spc and 17 μg/ml of Cam, or 50 μg/ml of Amp, 50 μg/ml of Spc, 17 μg/ml of Cam, and 30 μg/ml of kanamycin (Kan). The reversion frequencies were calculated as the ratios of the number of Kan^R^ colonies to the total number of colonies on Amp+Spc+Cam plate. The relative reversion frequency was then determined as the ratio of reversion frequencies from one pair of identical bacteria cultures, in which *Kan*^*R*^ transcription was induced with IPTG either in the presence or in the absence of deaminase expression. To confirm that the Kan^R^ colonies really were a result of DNA deamination, the pTAC-Kan plasmids from multiple colonies were purified and the reversion of the stop codon confirmed by DNA sequencing.

### CDA assays using *rpoB* as the reporter

An optimized protocol of a widely used assay to measure polynucleotide CDA activities^35^ was used. Individual pASK expression vectors of the invertebrate deaminases were transformed separately into UDG-deficient BH156 *E. coli*. Four ml cultures of LB medium containing 100 µg/ml Amp, 17 µg/ml Cam, and 0.3 µg/ml AHT were inoculated with single bacterial colonies. Cultures were grown for 24 h at 37 °C 230 rpm, aliquots were plated onto LB agar plates containing 100 µg/ml Amp or 100 µg/ml rifampicin, and grown at 37 °C overnight. Colonies were counted and the reversion frequencies were calculated as the ratio of the numbers of Rif^R^ colonies to the numbers of Amp^R^ colonies from the same culture. Cultures expressing HsAID or containing the empty pASK expression vector served as positive and negative controls, respectively. At least eight cultures per expression construct were tested. To determine the identity of mutations in the rpoB gene from rifampicin-resistant colonies, colony PCRs were performed from 24 individual colonies using Phusion polymerase and the rpoBF/rpoBR primer pair. PCR products were gel purified and subjected to sequencing using the rpoBF primer. Mutations were identified by comparing with the WT sequence (Genbank acc. CP024859.1, nt 2321219-2321797).

### Protein expression of invertebrate deaminases in *E. coli*

For monitoring the expression level of SpAIDLX/LaAIDLX in the deaminase assays, pASK_V5-SpAIDLX/LaAIDLX were transformed into UNG-deficient BH156. Individual colonies were grown in LB broth (containing 50 μg/ml of Amp, 50 μg/ml of Spc, and 17 μg/ml of Cam) until the optical density at 600 nm was 0.3. IPTG (1 mM) and AHT 0.2 (μg/ml) were added to the culture to induce protein expression. After incubation for 3 h (37 °C, 200 rpm), cultures were pelleted and washed with PBS. The pellets were lysed in 2x SDS sample buffer, diluted ten-fold, and separated on a pair of 10% SDS-PAGE gels. While one gel was stained with Coomassie blue to assess whether equal amounts of proteins were loaded, the other gel was transferred to PVDF membranes by semi-dry transfer and V5-tagged deaminases were detected using a mouse anti-V5 primary antibody (Abcam, ab27671) and a horseradish peroxidase coupled rabbit-anti mouse secondary antiserum (Jackson ImmunoReasearch). Chemiluminescent signals were visualized using the Immobilon Western Chemiluminescent HRP Substrate (Millipore) and X-ray film or a ChemiDoc Touch (BioRad) imaging system. Uncropped images of all stained protein gels and western blots are shown in Supplementary Fig. 5.  All bacterial expression experiments were performed in duplicate.

### Bioinformatics

Sequence comparisons to the sea urchin genome were done using BLAST, BLASTP, or BLASTN at https://blast.ncbi.nlm.nih.gov/Blast.cgi or www.echinobase.org using either standard parameters or with low complexity filter turned off. Multiple sequence alignments were performed using CLUSTALW and optimized manually based on predicted secondary structural features. Phylogenetic and molecular evolutionary analyses were performed using MEGA (version 7)^36^.

### Data availability

Sequence data that support the findings of this study have been deposited in Genbank with the following accession codes: *SpAIDL1* (MH106904, MH106887, MH106888, MH106889, MH106890, MH106891), *SpAIDL2* (MH048921, MH048922, MH048923, MH048924, MH048925), *SpAIDL3* (MH106892, MH106893, MH106894, MH106895, MH106896, MH106897), *SpAIDL4a* (MH106905, MH080287, MH080288, MH080289, MH080290), *SpAIDL9* (KY241384, KY241385, MH106898, MH106899, MH106900, MH106901, MH106902, MH106903), *LaAIDL1* (MH106906), *LaAIDL2* (MH106907).

All other data that support the findings of this study are available from the corresponding author upon reasonable request.

## Electronic supplementary material

Supplementary Information

Peer Review File
