# Supplementary material for: AID/APOBEC-like cytidine deaminases are ancient innate immune mediators in invertebrates
Source: Nat Commun. 2018 May 16;9:1948. doi: 10.1038/s41467-018-04273-x (PMC5956068; doi:10.1038/s41467-018-04273-x)
Supplement: Supplementary file 1 — Supplementary Information [file 41467_2018_4273_MOESM1_ESM.pdf]

Supplementary Information

AID/APOBEC–like cytidine deaminases are ancient innate immune mediators in invertebrates

Liu et al.

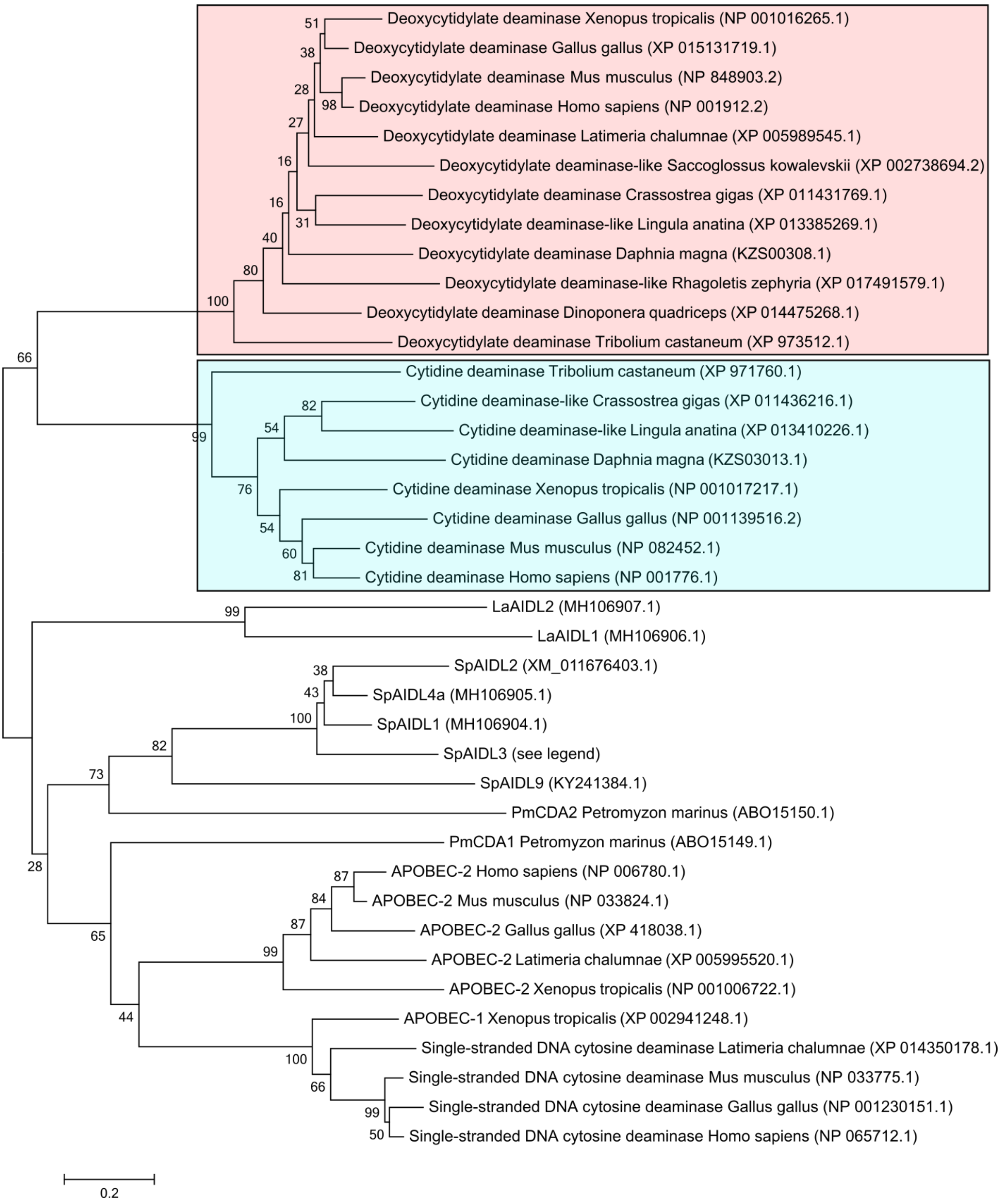

Supplementary Figure 1. Phylogenetic relationship of vertebrate and invertebrate deaminases. The amino acid sequences of the core region of the cytidine deaminases (CDAs), deoxycytidine-5-monophosphate deaminases (dCMP DAs), APOBEC2s, and AIDs from selected species were aligned using CLUSTALW, and alignments were manually adjusted based on secondary structure predictions. A Neighbor joining tree was calculated using MEGA with 1000 bootstrap replications. The numbers at each branch point indicate the bootstrap values for each branch point. The clusters of CDAs and dCMP DAs are highlighted in red and blue respectively. The SpAIDL3 sequence is derived from the *S. purpuratus* genome v3.1 Scaffold300 (nt 227523-227552 joined to nt 231537-232087). Note that despite the confusing nomenclature, PmCDA1 and PmCDA2 are the lamprey AID-like proteins and not monomeric cytidine deaminases (CDAs).

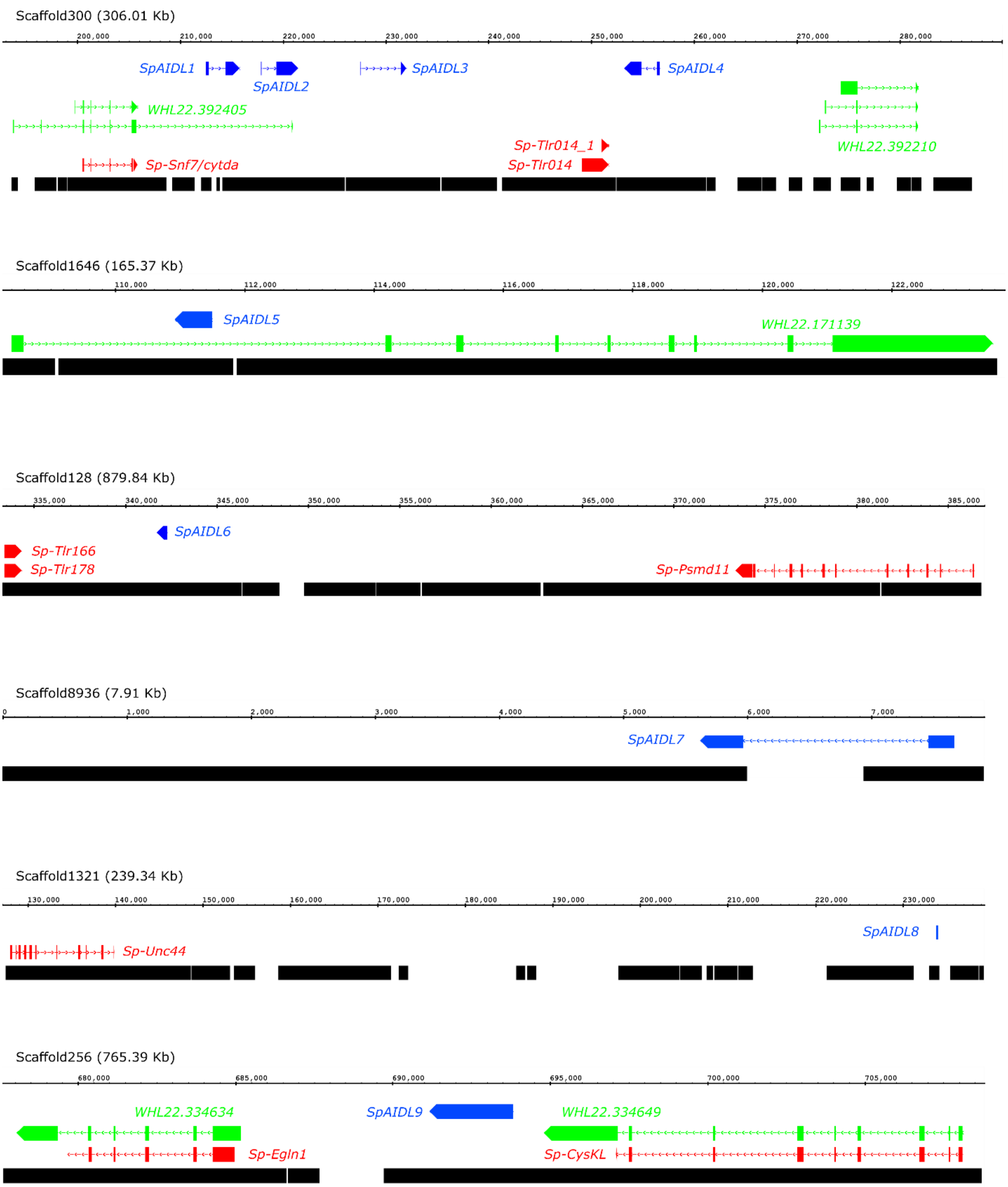

Supplementary Figure 2. Schematic representation of the sea urchin *SpAIDL* gene loci. The genomic location of each of the *SpAIDL1-9* genes is shown in blue, and the neighboring genes in the current *S. purpuratus* genome annotation and transcripts derived from a recent transcriptome analysis [24] are shown in red or green, respectively. The individual sequence contigs within each Scaffold are shown as black bars. All genomic coordinates and scaffold numbers refer to the version 3.1 of the *S. purpuratus* genome (www.echinobase.org). Note that there is an updated and more complete assembly available (version 4.2) but the gene annotation for this version is not publicly available. Genomic coordinates for the pseudogenes: *SpAIDL5* nt111506-110925, *SpAIDL6* nt342326-341748, *SpAIDL7* nt5971-5623 and nt 7666-7458, *SpAIDL8* 234136-233870)

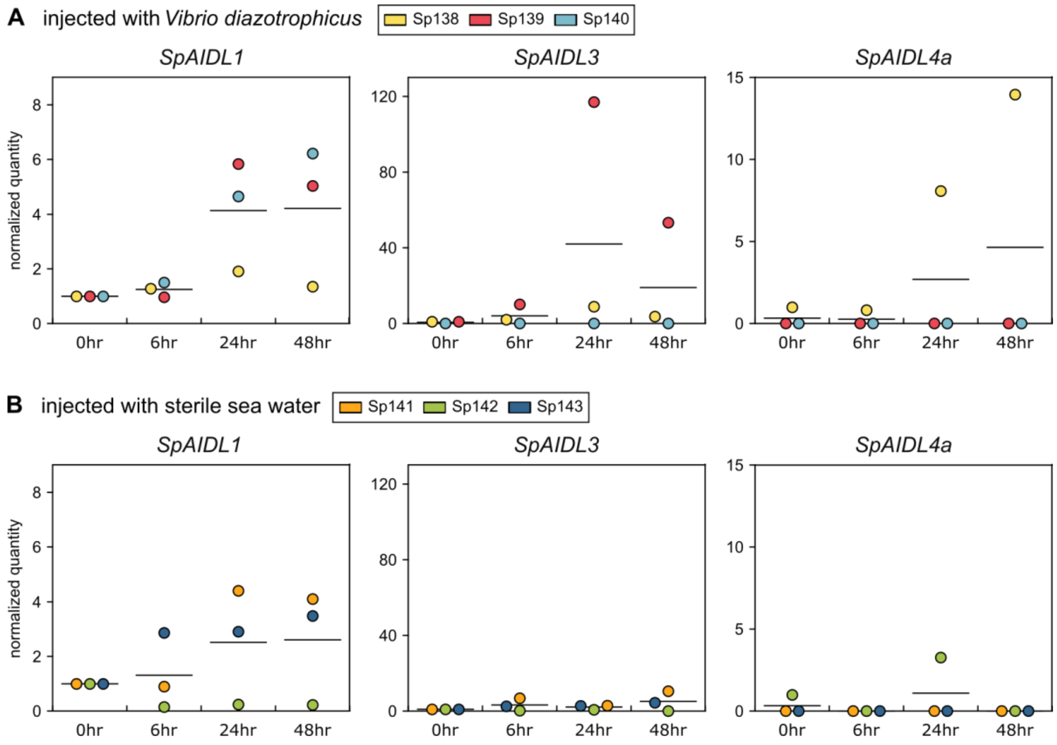

Supplementary Figure 3. Expression of *SpAIDLs* in *S. purpuratus in a bacterial infection model*. Six individual *S. purpuratus* were injected with either (**A**) live *V. diazotrophicus* (Sp138, 139, and 140) or (**B**) sterile sea water (Sp141, 142, and 143). The expression levels of *SpAIDL1, SpAIDL3, SpAIDL4a,* and *18S* in coelomocytes at the indicated time points were measured by RT-qPCR with two technical replicates per sample. The expression levels were first normalized to the amount of *18S* and then normalized to the expression at t=0 for each transcript for each urchin. Each data point is the average of two technical replicates. The horizontal bars indicate the mean.

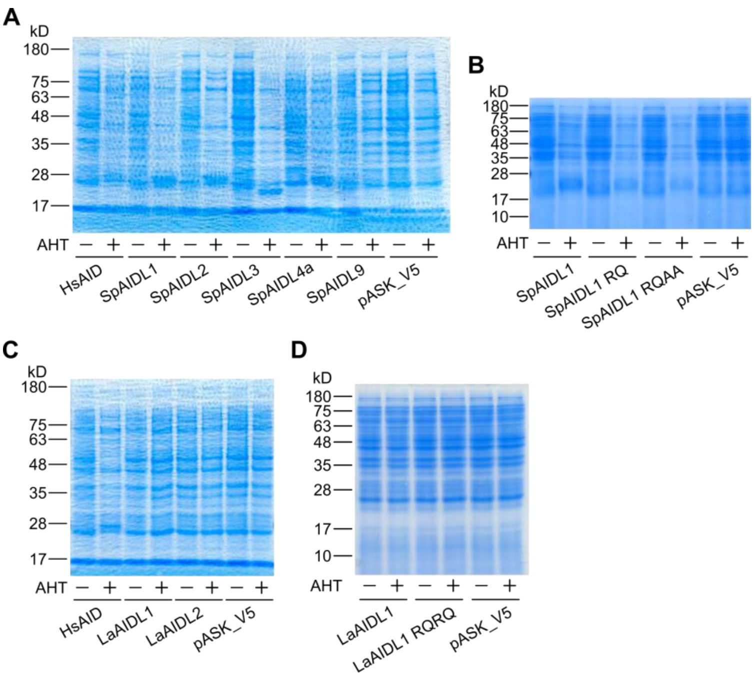

Supplementary Figure 4. Loading control for SpAIDL expression levels in *E. coli*. UNG-deficient BH156 *E. coli* strains containing one of the indicated deaminase expression vectors, pASK_V5-SpAIDL1, 2, 3, 4a, 9, LaAIDL1, or 2, were grown and the expression of the deaminase (+) was induced with anhydrotetracyclin (AHT) for 3 hrs, or not (-). Aliquots of each culture were separated on a 10% SDS/PAGE gel and proteins were visualized by staining with Coomassie blue. The identity of each sample is indicated below the lane. The comparable intensity of staining between each lane indicates that each sample contained comparable amounts of protein. The predicted sizes for V5-tagged HsAID, SpAIDL1, 2, 3, 4a, and 9 are 25. 4 kD, 24.0 kD, 23.5 kD, 22.3 kD, 23.6 kD, and 24.6 kD, respectively.

Fig. 3D

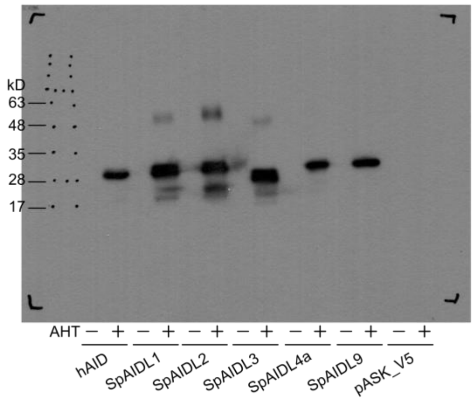

Fig. 3E

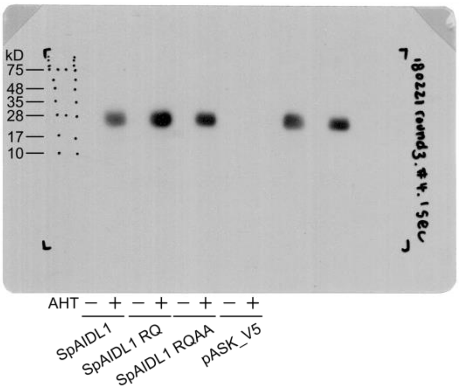

Supplementary Fig. 4A

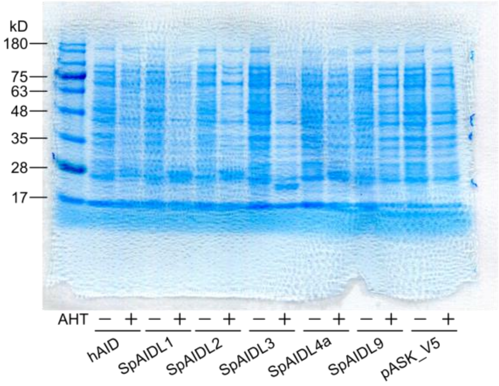

Supplementary Fig. 4B

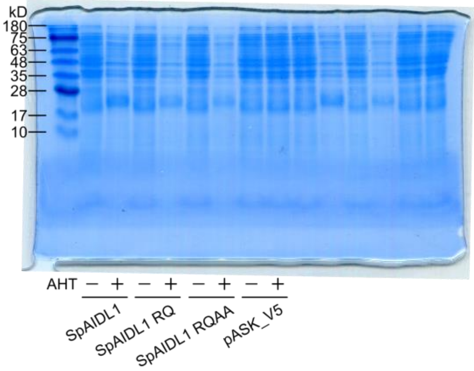

Fig. 5G

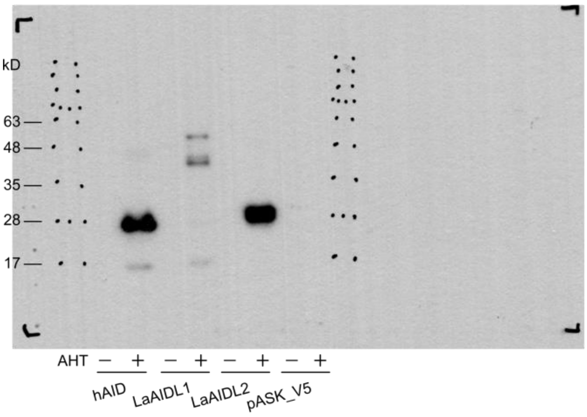

Fig. 5H

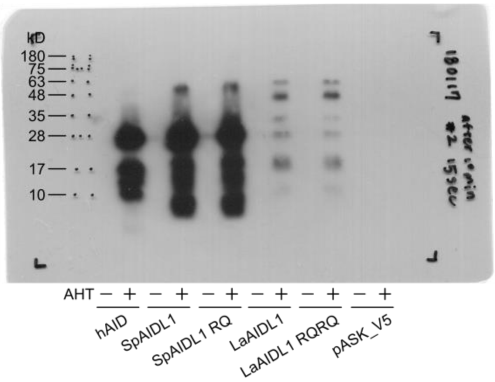

Supplementary Fig. 4C

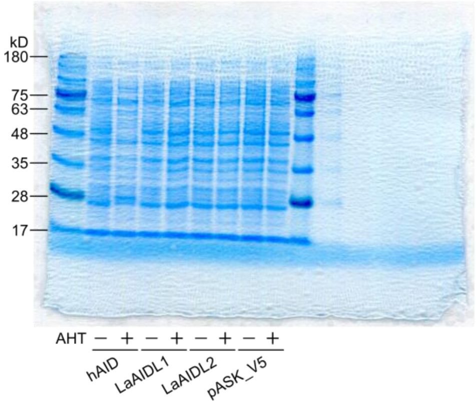

Supplementary Fig. 4D

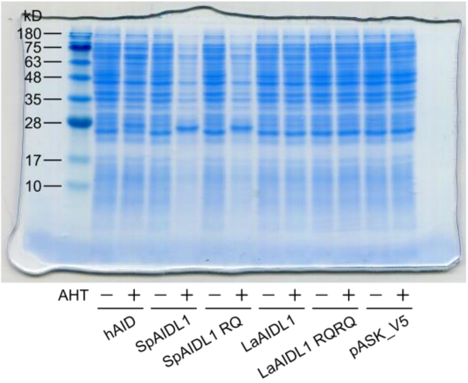

Supplementary Figure 5. Uncropped images of all Western blots and protein gels. It is indicated which for Figure panel each scanned image served as the source.

Supplementary Table 1.
Primer sequences for generating and analyzing plasmids.

Target	Primer	Sequence
SpAID1	SPA1FLF	GACTACTAGTATGCAGCACGACTTCCTCT
	SPA1FLR	TGACCTCGAGTTAAATTTTCATCACTCTGA
	SPA1FLRN	AGTCCTCGAGAATTTTCATCACTCTGAAGTTAGG
	SPA1H85RE87	AGGTAGCGGAACCGCacgtGCCcaaGAGATGATACTGAA
	SPA1H85RE87	TTCAGTATCATCTCttgGGCacgtGCGGTTCCGCTACCT
	SPA1CCAA-P	TTTCTACGTCTCCTCCGGGAG
	SPA1CCAAB	ACTCTCCAAAAAATGGATAGCTCCACTAGCAGGAGATAAGACTGTGAA
SpAID2	SPA2FLF	GACTACTAGTATGTATCAAGACAAGGTCA
	SPA2FLR	TGACCTCGAGCTAATTATATTTATTTATCTTTATC
	SPA2FLRN	AGTCCTCGAGATTATATTTATTTATCTTTATC
SpAID3	SPA3FLF	GACTACTAGTATGGATCACGACTACCAC
	SPA3FLR	TGACCTCGAGTTAATAAATCTTCATCAC
	SPA3FLRN	AGTCCTCGAGATAAATCTTCATCACTTTGAAGTTAGGG
SpAID4a	SPA4AFLF	GACTACTAGTATGCATCACGACGCCCCTCA
	SPA4AFLR	TGACCTCGAGTTAAGAAATCTTTATCACTCTG
	SPA4AFLRN	AGTCCTCGAGAGAAATCTTTATCACTCTGAAGTTAGG
SpAID9	SPA9CSFN	CATTACTAGTTATGAAGCAACGAGTGT
	SPA9C-XhoR	CAATCTCGAGATTCACCATCTTGACCTTTCAG
	SPA9FLRN	AGTCCTCGAGGGACTCAATGTGGAGCTTCG
	SPA9S3FT	GAGGGCAAAAAATGGCTTTTTATGAAGCAACGAGT
	SPA9S3FB	ACTCGTTGCTTCATAAAAAGCCATTTTTTGCCCTC
LaAID1	LaA1-FLFSP	AAAACTAGTTAACGAGGGCAAAAAATGGCTCACTCTACCAGT
	LaA1-FLRXH	TGACCTCGAGTTACTGATACTGCATCCACC
	LaA1-FLRX	TGACCTCGAGCTGATACTGCATCCACC
	LaA1H81RE83	GGTATCGCATTCATTTGCGCGCGGACACCGGGTGTATT
	LaA1H81RE83	TTACCTAGAAAACCGAGGTGTC
	LaA1H298RE3	CTTATCGCATGTTTTTGGGCACGCGTTTCATTAAAGCC
	LaA1H298RE3	TTACCTAAAAAAGCGCCAAG
LaAID2	LaA2-FLFSP	AAAACTAGTTAACGAGGGCAAAAAATGAATTCAGTGAGTGCC
	LaA2-FLRXH	TGACCTCGAGACCCTGCAACTCAGTAAAGTA
	LaA2-FLRX	TGACCTCGAGATTCTTATTTTTCTTCTCATCC
HsAID	hAIDFS	GATAACTAGTCTCTTGATGAACCGGAGGAA
	hAIDRX	GACTCTCGAGTCAAAGTCCCAAAGTACGAAA
	hAIDFLRX	AGTCCTCGAGAAGTCCCAAAGTACGAAATG
	hAID-A2DT	ACGAGGGCAAAAAATGGACAGCCTCTTGATGAAC
	hAID-A2DB	GTTCATCAAGAGGCTGTCCATTTTTTGCCCTCGT
V5 tag	pASK_V5F	CTAGCCATCATCTCGAGTTAGGAAGTGAAGGTAAGCCTATCCCTAACCCTCTCCTCGGTCTGGATTCTACGTAAG
	pASK V5R	TCGACTTACGTAGAATCCAGACCGAGGAGAGGGTTAGGGATAGGCTTACCTTCACTTCCTAACTCGAGATGATGG
Kan^R	KanSR	GTAATCGCAACATCCGCATT

Supplementary Table 2.

Primer sequences for the genomic sequence analysis, 5'/3' RACE, and *rpoB* gene mutation analysis

Target	Primer	Sequence
SpAID1	SPA1CF1	ATGGCATCGATGCAGCACGACTTCC
	SPA1CR	GCTCGATTCCACTGGTCTTTCAA
SpAID2	SPA2CF1	ATGGCATCGATGTATCAAGACAAG
	SPA2CF3	CCGACAGTGTGTAAAGGGCT
	SPA2CR1	CTAATTATATTTATTTATCTTTATCAC
	SPA2CR2	CTCAATCTCGCTGGCCTACC
SpAID3	SPA3CF1	CATCGATGGATCACGACTAC
	SPA3CR1	TTAATAAATCTTCATCACTTTGAAGTTA
SpAID4a	SPA4CF1	ATGGCATCGATGCATCACGACG
	SPANCR1	TTAAGAAATCTTTATCACTCTG
SpAID9	SPA9CF	ATGGCATTTTATGAAGCAACGA
	SPA9CR	ATTCACCATCTTGACCTTTCAG
	SPA9CR1	AGAGCTCGCCAATTTCCGGTC
	SPA9CR2	CGGCAATTTTGTCCGCAACCC
	SPA9CF1	GACCGGAAATTGGCGAGCTCT
	SPA9CF2	GGGTTGCGGACAAAATTGCCG
pJET 1.2	pJET1.2 forward	CGACTCACTATAGGGAGAGCGGC
	pJET1.2 reverse	AAGAACATCGATTTTCCATGGCAG
rpoB gene	rpoBF	TTGGCGAAATGGCGGAAAACC
	rpoBR	CACCGACGGATACCACCTGCTG

Supplementary Table 3.

Primer sequences for the gene expression analysis

Target	Primer	Sequence
SpAID1	SPA1Q2F	CGGACGGACAAATGCACAC
	SPA1Q2R	CCACATCAGTCCCTTCTCCC
SpAID2	SPA2Q1F	CGACGACGAGTTCATGGCTA
	SPA2Q2F	TGGCTACATGTCCGAGATCC
	SPA2Q1R	TATTTCTCCGTCTGGTGGCG
	SPA2Q2R	TCCGACTTTGTAACCATTTCAGT
SpAID3	SPA3Q1F	ATGGACACCGACTTCCCAAC
	SPA3Q1R	CTACCTTCGGATTTGGGGCA
SpAID4a	SPA4AQ1F	CGTCTCCTCCGAGAAAAGGG
	SPA4AQ1R	TTCTTTGTGCTAGCGGCGTA
SpAID9	SPA9Q2F	AGCCGTCTATGCACTGGAAC
	SPA9Q2R	AAGACCCGTTCCGATTCACC
LaAID1	LaA1-F1	CACGCCGAAATGAATGCGAT
	LaA1-QR	TGCATGGGGACCAATTCAGG
LaAID2	LaA2-QF	CGACCGACATCATGGACACT
	LaA2-R1	AAGGAGCGAGGCAACTTCTC
18S	18SF	CAGGGTTCGATTCCGTAGAG
	18SR	CCTCCAGTGGATCCTCGTTA
SpIL17-IX	IL17-IX-qPCRF	CAATCAGGAGCCTCTCRAGT
	IL17-IX-qPCRR	AGGGTTAATACAATCACGGCAC
LaRPL39	LaRPL39-QF	TGGGACAGATTGGAGCAAAAC
	LaRPL39-QR	GCCATGTTCGGGATCTGTTG
