# Supplementary material for: AID/APOBEC-like cytidine deaminases are ancient innate immune mediators in invertebrates
Source: Nat Commun. 2018 May 16;9:1948. doi: 10.1038/s41467-018-04273-x (PMC5956068; doi:10.1038/s41467-018-04273-x)
Supplement: Supplementary file 2 — Peer Review File [file 41467_2018_4273_MOESM2_ESM.pdf]

Reviewers' comments:

Reviewer #1 (Remarks to the Author):

The manuscript “AID/APOBEC–like cytidine deaminases are ancient innate immune mediators in invertebrates“ represents a very interesting glimpse into the evolution of the AID/APOBECs, a gene family of DNA and RNA editors. The Authors report the identification of bona fide homologs of the AID/APOBECs in sea urchin and lingual, a brachiopod, with a high similarity to the vertebrate AID/APOBECs with regards to primary structure and domain organisation. Moreover, they correlate their expression to the function of the immune system, which clearly reminisce the role of AID, a vertebrate deaminase responsible for all the antigen-driven antibody diversification processes. This report therefore traces back the link between this gene family and the immune function prior to deuterostomes and hints that (foreign) DNA-targeting molecules were present even before the rise of adaptive immunity. All this is clearly a valuable entry to understand the evolution of the immune system, with the potential to interest readers involved neither in AID/APOBEC research nor in immunity.

Comments:
- The presence of uncharacterized deaminases closer to the AID/APOBECs than to other deaminase groups had been reported in invertebrates (Hibino 2006, Severi 2011). How these deaminases compare to the reported ones?
- the DNA mutator assays are compelling. Yet, given the poor induction of a mutator phenotype in bacteria, it would be wise to add catalytically inactive mutants of the more active deaminases (SpAIDL1 and LaAIDL1).
- the Authors note that the lack of activity observed for many of the deaminases could be due to the non-physiological temperature used throughout the mutator assays. Have they tried to induce the expression of these deaminases at lower temperature? Reduction of the assay temperature has increased the induced mutator phenotype observed in other studies.
- statistics: an ANOVA test might be better suited to compare the induction of a mutator phenotype.
- another possible explanation for the lack of editing activity for some of the deaminases lies in the mutator assay used: since it is based on the reversion of a single point mutation (L49P) of the Kanamycin resistance gene, the results could be heavily affected by the nucleotide sequence context of the mutation. In these respects other types of reversion assays (eg the rifampicin-resistance assay) are less constrained by the sequence context preference of the tested mutators (as a bonus, sequencing of the reverted bacterial colonies would be informative of the sequence context preference). This option should be at least discussed in the text.
_ very minor: it might be more straightforward if the use of positive and negative control plasmids is mentioned earlier in the description of the mutator assay, as -from the way it is written- it sounds as these were controls for the sequencing (and a negative control for the sequencing in this assay is impossible).
- V5-tagged versions of the deaminases have been used to evidence their expression in bacteria. These figures are ambiguous, as it seems that the mutator assays and the western blots are part of the same experiment, when in fact derive from different constructs (are V5-tagged deaminases able to induce a mutator phenotype?).
- It might be worth expanding the discussion to include some speculation on the similarities/differences among invertebrate and vertebrate immune systems and their relation to the AID/APOBECs.
- Most of the manuscript depicts these deaminases as AID-like (starting from the name used), yet several parts suggest that their function might be more similar to that of the APOBEC3 (a successful test of their ability to interfere with mobile elements could provide a definitive answer!). For example, it is suggested that the lack of induction of a mutator phenotype by some of these deaminases might point to a deamination-independent mechanism of action. While it is true that some APOBECs have such deamination-independent activity, all foreign-DNA targeting AID/APOBECs show an enzymatic activity much higher than that of AID. This does not fit well with

the data shown. Can the authors speculate about this aspect?

Reviewer #2 (Remarks to the Author):

This is an interesting manuscript that describes a well done genomics and functional study that provides convincing evidence for the presence of an AID/APOBEC system in two invertebrate species. The description is of notably broad general interest and particularly well written. The shortcoming of the work, which these authors can remedy easily, is an insufficient description of the broader significance of this finding, perhaps one of the most striking instances of how the adaptive immune system acquires its unique complexity by co-opting and integrating the functions of a remarkable number of otherwise unrelated processes to form unique mechanisms for creating and selecting somatic variation in B cell antigen receptors. Although certainly not a requirement to get across the primary message of this work, it would be of interest to see if constructs derived from the invertebrate AID/APOBECs could rescue mutant cell lines in which APOBEC/AID function has been compromised.

Reviewer #3 (Remarks to the Author):

In this study, Liu et al. identified putative AID-like genes in two invertebrates, sea urchins and brachiopods for the first time. The authors analyzed their enzymatic activity in bacterias and the up-regulation of these genes after pathogen challenge. The observations look interesting. However, the results and conclusions are not convincing. For instance, the sequence similarity of these genes identified by the authors to vertebrate AIDs is not significant. Also, their enzymatic activity is apparently lower than human AID. Moreover, the evidence that these genes play some roles in immunity against pathogens is not provided.

Major

1. page 4, extended figure 1 and Fig. 3 - “the SpAID proteins cluster with vertebrate AID and not the distantly related cytidine deaminases (CDAs) or deoxycytidine-5-monophosphate deaminases (dCMP DAs) that share a very similar catalytic motif (Extended Data Fig. 1).” However, the bootstrap value on that node of SpAIDs with vertebrate AIDs (45) is not significant. Moreover, the enzymatic activities of SpAIDLs are quite lower than that of human AID. Therefore, it would be hard to believe that SpAIDLs are orthologs of vertebrate AID from this observation.

2. Related to comment 1 - to convincingly assess the enzymatic activity of SpAIDLs, certain mutants that are defective in their (putative) catalytic domains should be used.

3. page 7 and Fig. 4 - similar to comment 1, the bootstrap value on the node of LaAIDLs with vertebrate AIDs (28) is not significant.

4. Abstract - the authors speculate that these AID-like genes play some roles in immunity in these invertebrates. In fact, the authors demonstrated that these genes are up-regulated upon pathogen challenge. However, the direct evidence suggesting that these AID-like genes “actually” function as immunity against pathogens is not provided.

Minor

As the authors may know vertebrates encode sole AID gene. Why sea urchins and brachiopods encode multiple AID genes?

First we would like to thank all three reviewers for recognizing the relevance and importance of our work and appreciate the insightful comments. The suggested additional experiments we performed helped to strengthen the main point of our manuscript. Furthermore, the suggested changes to the text clearly emphasize the broad significance of our findings. Below are the point-by-point responses to each reviewers' comments:

Reviewer #1:

- The presence of uncharacterized deaminases closer to the AID/APOBECs than to other deaminase groups had been reported in invertebrates (Hibino 2006, Severi 2011). How these deaminases compare to the reported ones?

We would like to thank the reviewer for pointing out these two references. While we were aware of the Hibino et al. (2006) paper, we actually did not recall that the phylogenetic tree in the Severi & Conticello (2011) paper also contained some un-annotated putative invertebrate AID/APOBEC-like proteins. The deaminases discovered in our study are different from those described by Hibino et al. (2006) ["…phylogenetic analysis indicates that none of them belong to the AID/APOBEC gene family…"] and Severi & Conticello (2011). Interestingly, in a recent scan of invertebrate genome/transcriptome databases for homologs of our LaAIDL1 protein, we also identified one of the predicted *C. gigas* proteins identified by Severi et al. (data not shown). We would like to emphasize that we did not specifically set out to discover novel AID/APOBEC-like deaminases in invertebrates based on sequence motifs but rather "stumbled" over them during an unrelated transcriptome data analysis. This work is thus the first report of invertebrate deaminases in which the predicted transcripts were experimentally validated using RT-PCR, rather than relying solely on bioinformatics data. We also do not have any data regarding the enzymatic activity of the previously reported candidates, but we will perform such studies in the future.

In the revised manuscript, we have included a sentence describing these previous reports, stating that our deaminases differ from those predicted proteins, and added the respective publications to our reference list.

- the DNA mutator assays are compelling. Yet, given the poor induction of a mutator phenotype in bacteria, it would be wise to add catalytically inactive mutants of the more active deaminases (SpAIDL1 and LaAIDL1).

We agree with the reviewers that demonstrating the functional role of the putative active sites within the deaminases is an important aspect of understanding observed mutator phenotypes. Therefore, we generated SpAIDL1 and LaAIDL1 mutants in which the HxE motif of the actives site(s) were mutated to RxQ (these mutations have been reported to dramatically reduce the activities of all vertebrate AID/APOBEC deaminases [1]). The mutagenic activities of these altered proteins were subsequently tested in both of the *E. coli* based deamination assay (Figs. 3C, 4D, and 4F). While both mutants showed reduced activity compared to the respective wild-type enzymes, some residual activity remained for the SpAIDL1 mutant. We therefore mutated the remainder of the zinc-coordinating residues within the SPCxxC motif to SPAxxA (SpAIDL1RQAA). No catalytic activity was evident in this mutated protein (Fig. 3C). The *rpoB* mutation assay highlighted additional differences between the wild-type, catalytic enzymes and the mutants with modified active sites. Mutations in the *rpoB* gene confer rifampicin resistance that is scored in this assay. Sequencing the *rpoB* genes of rifampicin-resistant colonies revealed clear differences in the mutation spectra of each of the active site mutants compared to the parental enzymes (Extended Data Fig. 5). Taken together this strongly supports our conclusion that SpAIDL1 and LaAIDL1 are able to deaminate ssDNA in a manner similar to most vertebrate AID/APOBEC proteins.

We have modified and added the respective figures and corresponding text to the revised version of our manuscript.

- the Authors note that the lack of activity observed for many of the deaminases could be due to the non-physiological temperature used throughout the mutator assays. Have they tried to induce the expression of these deaminases at lower temperature? Reduction of the assay temperature has increased the induced mutator phenotype observed in other studies.

We thank the reviewer for this great suggestion as the lack of activity for some of our novel invertebrate deaminases has been a little puzzling. The central point of the current manuscript is to demonstrate the existence of invertebrate AID/APOBEC-like deaminases with activities similar to those of their vertebrate relatives, and hence with limited time available for revisions we decided to focus first on the experiments centered on SpAIDL1 and LaAIDL1 that support this main conclusion. Nevertheless, to assess whether lower temperatures would increase or reveal activity for SpAIDL9 (one of the enzymes that was apparently inactive or only minimally active in our Kanamycin resistance assay), we performed

a pilot experiment using rifampicin resistance as the read-out in which the *E. coli* were grown and protein production was induced at 25°C. Comparisons between the enzyme and the empty vector negative control, revealed no striking increase compared to the same assay performed 37°C (data not shown). While a more detailed exploration of the temperature dependence of all invertebrate AID/APOBEC-like proteins is outside the scope of this study, it clearly is one of our lines of investigation in the future in particular once we are able to produce recombinant enzymes for biochemical studies.

- statistics: an ANOVA test might be better suited to compare the induction of a mutator phenotype.

We share the reviewers concern about the identifying and using the appropriate statistical test for our dataset, and hence we consulted with a biostatistics expert (Dr. Pei-Chien Tsai) in our department. A one-way ANOVA test is relevant for comparing the differences among groups, considering all possible pairs of groups (here, deaminases); but we are only interested in testing whether each deaminase differs compared to the control group (the empty expression vector, pASK). Furthermore, the ANOVA test (just like the T test) considers a normal distribution, but (if at all) our dataset is derived from truncated normal distributions, since values below zero are impossible in our assay. Given the small number of N in our datasets (ranging from 8-16) and some apparent "outliers" (with unexplainably higher mutation/reversion frequencies) we now decided to use a non-parametric rank-based statistical test instead (that compares the medians rather than the means), the Willcoxon rank sum test (also called Mann-Whitney U test). To directly address the reviewers request, we also performed a one-way ANOVA test (using a post-hoc Dunnett's test) and, in this case, only the difference between HsAID and pASK appeared statistically significant ($p<0.05$). Although we were initially concerned about this (we did not know which statistical test we should actually believe), our *rpoB* gene sequence analysis revealed clear differences between the mutations introduced by SpAIDL1, LaAIDL1, and pASK. This provides additional direct biological evidence for mutator activities of the enzymes that are independent of mathematically determined p-values.

- another possible explanation for the lack of editing activity for some of the deaminases lies in the mutator assay used: since it is based on the reversion of a single point mutation (L49P) of the Kanamycin resistance gene, the results could be heavily affected by the nucleotide sequence context of the mutation. In these respects other types of reversion assays (eg the rifampicin-resistance assay) are less constrained by the sequence context preference of the tested mutators (as a bonus, sequencing of the reverted bacterial colonies would be informative of the sequence context preference). This option should be at least discussed in the text.

We would like to thank the reviewer for this great idea as it helped to provide additional support for the DNA deaminase function of SpAIDL1 and LaAIDL1 when expressed in *E. coli*. We now established the deaminase assay without the Kanamycin resistance reporter plasmid using mutations of the endogenous *rpoB* gene instead. Single point mutations in a variety of Cs within the *rpoB* gene confer rifampicin-resistance to *E. coli* [2]. The data obtained with this assay largely confirm the results of the Kan^R assay; SpAIDL1 and LaAIDL1 show deaminase activity compared to the negative control (pASK) (Figs. 3B and 4E). Importantly, we also determined the mutation patterns within the *rpoB* gene generated by the SpAIDL1 and LaAIDL1 deaminases and their active site mutants SpAIDL1 RQ, SpAIDL1 RQAA and LaAIDL1 RQRQ (Extended Data Fig. 5). The two main findings from this data set are (1) that these enzymes show altered preferences compared to both HsAID and the negative control, and (2) that mutating the active site residues alters the mutation patterns created by the enzymes. This strongly suggests that SpAIDL1 and LaAIDL1 indeed act as DNA deaminases in this *E. coli* assay and thus show properties similar to most vertebrate AID/APOBEC enzymes.

We modified and added the respective figures and added corresponding text to the revised version of our manuscript.

- very minor: it might be more straightforward if the use of positive and negative control plasmids is mentioned earlier in the description of the mutator assay, as -from the way it is written- it sounds as these were controls for the sequencing (and a negative control for the sequencing in this assay is impossible).

We changed the text according to the reviewer's suggestions.

- V5-tagged versions of the deaminases have been used to evidence their expression in bacteria. These figures are ambiguous, as it seems that the mutator assays and the western blots are part of the same experiment, when in fact derive from different constructs (are V5-tagged deaminases able to induce a mutator phenotype?).

We apologize for this confusion. Thus far we only tested the un-tagged proteins in the deaminase assays as widespread anecdotal evidence suggests that tags alter the activities of vertebrate AID/APOBEC deaminases. We felt that using tagged constructs for testing expression of the deaminases is a reasonable compromise to be able to compare expression levels of the different enzymes. For our future work utilizing recombinant SpAIDL1 and LaAIDL1 that are outside the scope of this manuscript, we will perform the *E. coli* based deaminase assay with the respective tagged proteins as an important control.

We now altered the main text and figure legends to try to make it very clear that the deaminase assay and the western blots are independent experiments.

- It might be worth expanding the discussion to include some speculation on the similarities/differences among invertebrate and vertebrate immune systems and their relation to the AID/APOBECs.

We agree that the discussion was rather short and minimalistic in the first version of this manuscript. This was largely due to the fact that the manuscript was directly transferred to Nature Communications from a different Nature journal without adjusting the text.

We now included an expanded discussion that addresses these points.

- Most of the manuscript depicts these deaminases as AID-like (starting from the name used), yet several parts suggest that their function might be more similar to that of the APOBEC3 (a successful test of their ability to interfere with mobile elements could provide a definitive answer!). For example, it is suggested that the lack of induction of a mutator phenotype by some of these deaminases might point to a deamination-independent mechanism of action. While it is true that some APOBECs have such deamination-independent activity, all foreign-DNA targeting AID/APOBECs show an enzymatic activity much higher than that of AID. This does not fit well with the data shown. Can the authors speculate about this aspect?

We thank the reviewer for raising this very important point: which of the different vertebrate AID/APOBECs do the invertebrate enzymes that we discovered here most closely resemble. To be quite frank, we have struggled since the beginning of this project with the naming of our novel proteins and have worried about the subconscious inferences that our naming will cause in the readers' brain. We

honestly do not currently know whether these enzymes mutate endogenous genes, endogenous transcripts, foreign genetic material, or whether they act in a cytosine deamination independent manner against retro-(viral)elements, or even in completely different yet to be discovered ways (like APOBEC2 and APOBEC 4, [3]). We now add a few sentences to the discussion to lay out which of these possibilities is (in)-consistent with our observations in light of what is currently known about the vertebrate AID/APOBEC-like deaminases. We also kept the names of our enzymes for now (as AID-like), but if the reviewers (or the editor) would like to suggest a more suitable name we would be more than happy to change our nomenclature.

Reviewer#2

-The shortcoming of the work, which these authors can remedy easily, is an insufficient description of the broader significance of this finding, perhaps one of the most striking instances of how the adaptive immune system acquires its unique complexity by co-opting and integrating the functions of a remarkable number of otherwise unrelated processes to form unique mechanisms for creating and selecting somatic variation in B cell antigen receptors.

We thank the reviewer for suggesting to emphasize the broader implications of our findings for our understanding of how a central feature of adaptive immunity in vertebrates, antigen receptor repertoire diversity, has evolved. We now added a section that is focused on this aspect to the discussion at the end of the manuscript.

-Although certainly not a requirement to get across the primary message of this work, it would be of interest to see if constructs derived from the invertebrate AID/APOBECs could rescue mutant cell lines in which APOBEC/AID function has been compromised.

Testing the function of the invertebrate AID/APOBEC-like deaminases in the context of $AID^{-/-}$ vertebrate cell lines is clearly on our list of future experiments. One reason why such studies were not prioritized for this initial characterization, is that our lab has observed in the past that even the most ancient vertebrate AID/APOBEC-like proteins, CDA1 and CDA from *Petromyzon marinus*, are not able to rescue AID-deficiency (unpublished data) despite being implicated as essential for the assembly of VLR antigen receptor genes. This process is thought to be mechanistically closely related to the AID-dependent immunoglobulin gene diversification mechanisms in jawed vertebrates. Thus, the inability of lamprey AID/APOBECs to complement jawed vertebrate AID function was rather surprising, but was

consistent with similar anecdotal evidence from other labs in the field. Hence, we felt that this assay is quite specific for jawed vertebrate AID (none of the vertebrate APOBECs can complement AID function) and that it would be extremely unlikely that the even more distantly related invertebrate AID-like factors could do so. Given the limited time for the revisions of this manuscript we decided to focus first on additional experiments characterizing the deaminase activities of the enzymes in more detail instead of further broadening the scope of this study.

Reviewer #3

In this study, Liu et al. identified putative AID-like genes in two invertebrates, sea urchins and brachiopods for the first time. The authors analyzed their enzymatic activity in bacterias and the up-regulation of these genes after pathogen challenge. The observations look interesting. However, the results and conclusions are not convincing. For instance, the sequence similarity of these genes identified by the authors to vertebrate AIDs is not significant. Also, their enzymatic activity is apparently lower than human AID.

We discussed these two points (low similarity AID, and low enzymatic activity) below (response #1).

Moreover, the evidence that these genes play some roles in immunity against pathogens is not provided.

Please see our response below (response #4).

Major:

1. page 4, extended figure 1 and Fig. 3 - “the SpAID proteins cluster with vertebrate AID and not the distantly related cytidine deaminases (CDAs) or deoxycytidine-5-monophosphate deaminases (dCMP DAs) that share a very similar catalytic motif (Extended Data Fig. 1).” However, the bootstrap value on that node of SpAIDs with vertebrate AIDs (45) is not significant. Moreover, the enzymatic activities of SpAIDLs are quite lower than that of human AID. Therefore, it would be hard to believe that SpAIDLs are orthologs of vertebrate AID from this observation.

To address the first point of this concern (low similarity):

We have improved the amino acid sequence alignments for building the phylogenetic tree using secondary structure predictions, which results in a more robust tree. These short (approximately 200

amino acids), rapidly evolving sequences are difficult to confidently analyze from an evolutionary perspective. Beyond the highly conserved catalytic motif, the evolution of these proteins is primarily constrained by secondary, rather than primary structure, which interferes with generating a robust alignment that accurately reflects the homology of amino acid resides. We now include a tree (Extended Data Fig. 1) in which the bootstrap value, based on 1,000 replications clearly supports distinct clades for the deoxycytidylate deaminases(dCMP_DA)/cytidine deaminases and the AID/APOBEC-like deaminases, and importantly, the SPAIDL proteins cluster together with one of the jawless-vertebrate AID homologs, PCDA2, that mediates the somatic assembly of VLR genes. During the course of this analysis, we have explored much of the potential alignment- and tree- space using several algorithms at both steps, and find that, even in the absence of robust support, several features remain constant among the trees. (1) The monophyly of each deaminase family is maintained; dCMP_DA, CDA, AID, and APOBEC proteins always form well-supported (bootstrap > 95) clades. Similarly, the SpAIDL and LaAIDL proteins always cluster in species-specific monophyletic clades. (2) The dCMP_DA/CDA and AID/APOBEC clades are always supported, although with lower support values. (3) Although the placement of the invertebrate AIDL proteins described here is somewhat dynamic, these proteins never form supported clades with the dCMP_DA/CDA proteins. Instead, the SpAIDL/LaAIDL proteins typically cluster at the base of the AID/APOBEC clade with levels of support that vary based on the alignments. The instability of this tree is not unexpected, given the long divergence time since the last common ancestor of sea urchins and vertebrates (about 550 million years ago), and rapid divergence driven by species specific host-pathogen interactions are a common feature of immune genes. We thus supplement these phylogenetic conclusions with the data from transcript localization experiments, functional assays, and the presence of conserved catalytic motifs that reflect the biology of the vertebrate AID/APOBEC proteins.

To address the second point of this concern (low enzymatic activity):

While the enzymatic activities SpAIDL1 are clearly lower than that observed for human AID, we do not take this as a strong argument against our hypothesis that SpAIDLs are members of the AID/APOBEC-family of deaminases. First, the bacterial deaminase assay, as it is currently utilized by the field, has been specifically optimized to quantify activity of the vertebrate AID/APOBECS and it is likely that this assay provides less than optimal conditions for the deaminases from marine invertebrates. In addition to the temperature differential discussed in the text, the physiological salt concentration of *E. coli* is significantly lower relative to the marine environment, which may affect protein folding or

activity. Alternatively, the invertebrate deaminases may have bind to unique target sequences that are absent from the reporter genes. Furthermore, the published data on vertebrate deaminases indicate that both AID and APOBEC1 are active mutators of DNA in *E. coli*. However, APOBEC1 is known to be a bona fide RNA-editing enzyme. Thus, activity in this assay (which serves as inclusion criterion for being termed AID/APOBEC-like deaminases) is as an established indication that enzymes can act on polynucleotide substrates. Observed variation in activity within this assay is thus not necessarily reflective of actual differences in specific activities within the native context. Lastly, our newly included analysis of mutations within the *rpoB* gene clearly indicates that SpAIDL1 and LaAIDL1 both induce mutation profiles that are distinct from the spontaneously arising background mutations (Extended Data Fig. 5). Generating these C->T/G->A mutations is dependent on active site residues that are strictly conserved between our invertebrate AID/APOBEC-like factors and all known AID/APOBEC-family members (Figs. 3C, 4D, and 4F).

The information from our additional experiments strengthen our conclusion that SpAIDLs and LaAIDLs are indeed AID/APOBEC-like polynucleotide deaminases have been added to the text/figures.

2. Related to comment 1 - to convincingly assess the enzymatic activity of SpAIDLs, certain mutants that are defective in their (putative) catalytic domains should be used.

We agree that testing potential active site mutants is a critical control. We now generated such mutants and tested their activities. For details please see our response to reviewer #1 and the respective changes we made to the text of the manuscript.

3. page 7 and Fig. 4 - similar to comment 1, the bootstrap value on the node of LaAIDLs with vertebrate AIDs (28) is not significant.

Please see our response to comment 1.

4. Abstract - the authors speculate that these AID-like genes play some roles in immunity in these invertebrates. In fact, the authors demonstrated that these genes are up-regulated upon pathogen challenge. However, the direct evidence suggesting that these AID-like genes “actually” function as immunity against pathogens is not provided..

While we agree with the reviewer that, thus far, there is no direct evidence that these invertebrate AID/APOBEC-like factors play a role in defense against pathogens (including a potential role in restricting retroelements that endanger genomic integrity), this remains the most parsimonious explanation of the function of our invertebrate SpAIDLs and LaAIDLs.

The gold standard for assigning an immune function to a specific gene is to genetically modify an organism or cell population such that expression of the respective factor is abolished and demonstrate a defective response against pathogens. While this is a relatively straightforward approach in model organisms, our invertebrates are "non-model" organisms in which such an experiment is currently impossible. Morpholino-based knock-down approaches are commonly used in *S. purpuratus* to study early stages of development (but are not applicable to the adult stage), and no lab cultures of *L. anatina* (let alone genetic manipulations) have been established thus far. Our conclusion of an immune function is thus built on several complementary, but indirect lines of evidence as laid out in the text. Direct measures of a role for the AIDL factors in immunity are the focus of our future work and go well beyond the scope of this manuscript, which is focused on the initial characterization of these factors. This line of reasoning is not unprecedented in the field. A role for the sea lamprey (*Petromyzon marinus*) AID/APOBEC-like deaminases, PmCDA1 and PmCDA2, in immune responses is widely accepted despite the lack of direct evidence. We remain open to the idea that some or all of the SPAIDLs and LaAIDLs have non-immune functions (like the "orphan" vertebrate APOBECs, APOBEC2 and 4, whose activities and biological functions are less well-defined). However, even given this case, the mere existence of AID/APOBEC family members prior to the emergence of vertebrate adaptive immunity has broad and far reaching implications (see comments of reviewer #2) for understanding how antigen receptor diversification processes evolved.

In an attempt to strengthen the link between the SpAIDL molecules and immune response, we quantified *SpAIDL* expression following injection of two different doses of poly IC (a viral mimic) into the coelomic cavity (data not shown). It is not known if this TLR ligand elicits an immune response in *S. purpuratus* coelomocytes and, unfortunately, our marker of immune activation (*SpIL17-9*) was not transcriptionally regulated in these experiments. It remains unknown whether *SpIL17-9* is upregulated exclusively during anti-bacterial responses or if the poly IC doses were too low to initiate a response. Hence, these new datasets are not interpretable at this time.

We now added a sentence to the discussion to indicate that our evidence for an immune function is only indirect, and that it is possible that the biological function of the SpAIDLs/LaAIDLs despite the similarity in terms of sequence and enzymatic activities is very different from that of their vertebrate counterparts.

Minor

As the authors may know vertebrates encode sole AID gene. Why sea urchins and brachiopods encode multiple AID genes?

This is a very interesting point that is similar to a point raised by reviewer #2. We not have a definitive answer, but plausible explanation include that the these factors represent either functionally redundant proteins that are expressed indistinct cell types or stages of differentiation, or that they act on distinct substrates. We currently favor the latter as this is consistent with the wide array of cellular targets edited by different members of the vertebrate AID/APOBEC family.

References

1. Papavasiliou, F. N. & Schatz, D. G. The activation-induced deaminase functions in a postcleavage step of the somatic hypermutation process. *J. Exp. Med.* **195,** 1193–8 (2002).

2. Harris, R. S., Petersen-Mahrt, S. K. & Neuberger, M. S. RNA editing enzyme APOBEC1 and some of its homologs can act as DNA mutators. *Mol. Cell* **10,** 1247–53 (2002).

3. Conticello, S. G. The AID/APOBEC family of nucleic acid mutators. *Genome Biol.* **9,** 229 (2008).

REVIEWERS' COMMENTS:

Reviewer #1 (Remarks to the Author):

The Authors have answered all concerns very effectively. A very nice manuscript.

Very minor comments:
- while it is common to spell out acronyms, the "activation induced cytidine deaminase (AID)/apolipoprotein B editing complex (APOBEC)" is quite misleading as RNA editing of apolipoprotein B editing is a very minor part of APOBEC physiology. It might be more sensible to just leave APOBEC as it is.
- Additional biological evidence is good indeed: "lies, damned lies, and statistics...", and I agree the Willcoxon test to be the most appropriate test. Having said that, I wonder why a normal distribution for the mutator assays would not be expected: it is true that values cannot be negative, yet normal distributions are auspicabile. Values on the 0 should be outliers if the assay is done correctly and not an indication of a truncated distribution (i.e. it would be impossible to assess the induction of the mutator phenotype if the negative controls give no revertants). Moreover the comparison is not just with the negative control but also with all other samples that fail to trigger a mutator phenotype.

Reviewer #2 (Remarks to the Author):

The authors have done an exemplary job of addressing the exensive comments of the reviewers and by conducting additional experiments and expanding the discussion have further improved an outstanding manuscript. I strongly urge that this important work proceed to publication.

Reviewer #3 (Remarks to the Author):

The revised manuscript and reply letter addressed my concerns satisfactory.

Point-by-point responses to the reviewers

<u>Reviewer #1 (Remarks to the Author):</u>

The Authors have answered all concerns very effectively. A very nice manuscript.

Thank you.

Very minor comments:

- while it is common to spell out acronyms, the "activation induced cytidine deaminase (AID)/apolipoprotein B editing complex (APOBEC)" is quite misleading as RNA editing of apolipoprotein B editing is a very minor part of APOBEC physiology. It might be more sensible to just leave APOBEC as it is.

We understand and share the reviewers' concern. But the *Nature Communications* guidelines require us to spell out the abbreviation at the first time we used it in the manuscript.

- Additional biological evidence is good indeed: "lies, damned lies, and statistics...", and I agree the Willcoxon test to be the most appropriate test. Having said that, I wonder why a normal distribution for the mutator assays would not be expected: it is true that values cannot be negative, yet normal distributions are auspicabile. Values on the 0 should be outliers if the assay is done correctly and not an indication of a truncated distribution (i.e. it would be impossible to assess the induction of the mutator phenotype if the negative controls give no revertants). Moreover the comparison is not just with the negative control but also with all other samples that fail to trigger a mutator phenotype.

We thank the reviewer for his insightful comments, and appreciate that the reviewer recognizes the strength of our additional biological evidence.

In our previous "response to reviewers" we wanted to point out that we simply do not know whether the distributions of the mutation frequencies are normally distributed or not. We agree with the comment that "negative outliers" should accumulate with zero values. Given that the value of our standard deviation (calculated assuming normality) is larger than the true value our experimental data a large part of the experimental data would theoretically expected to lie below zero. So, what we would be doing in essence is trying to fit a Gaussian curve for a dataset that should look like a truncated Gaussian curve. Hence we think that the Willcoxon test is a more appropriate method of analyzing the data.

When we conducted our initial experiments we asked: Does any of our deaminases have a higher mutagenic activity than the negative control? Thus we conducted the appropriate comparisons and our statistical analysis rejected the null hypothesis (mutator activity is the same than for the empty vector) for some of the samples. Now, from this analysis some seemingly "inactive" samples emerged. But these were not negative controls when we performed the experiments, and should thus not be treated as such afterwards for the analyses.

Reviewer #2 (Remarks to the Author):

The authors have done an exemplary job of addressing the exensive comments of the reviewers and by conducting additional experiments and expanding the discussion have further improved an outstanding manuscript. I strongly urge that this important work proceed to publication.

Thank you. We tried our best.

Reviewer #3 (Remarks to the Author):

The revised manuscript and reply letter addressed my concerns satisfactory.
